# Direct Observation of Ultrafast Lattice Distortions during Exciton–Polaron Formation in Lead Halide Perovskite Nanocrystals

**DOI:** 10.1021/acsnano.2c06727

**Published:** 2023-01-18

**Authors:** Hélène Seiler, Daniela Zahn, Victoria C. A. Taylor, Maryna I. Bodnarchuk, Yoav William Windsor, Maksym V. Kovalenko, Ralph Ernstorfer

**Affiliations:** † Fritz Haber Institute of the Max Planck Society, Faradayweg 4-6, 14195 Berlin, Germany; ‡ Physics Department, Free University of Berlin, Arnimallee 14, 14195 Berlin, Germany; § Laboratory for Thin Films and Photovoltaics, Swiss Federal Laboratories for Materials Science and Technology, Überlandstrasse 129, CH-8600 Dübendorf, Switzerland; ⊥ Institut für Optik und Atomare Physik, Technische Universität Berlin, Straße des 17. Juni 135, 10623 Berlin, Germany; ∥ Institute of Inorganic Chemistry, Department of Chemistry and Applied Biosciences, ETH Zürich, CH-8093 Zürich, Switzerland

**Keywords:** lead halide perovskites, nanocrystals, polaron formation, hot-phonon bottleneck, femtosecond electron diffraction, lattice dynamics

## Abstract

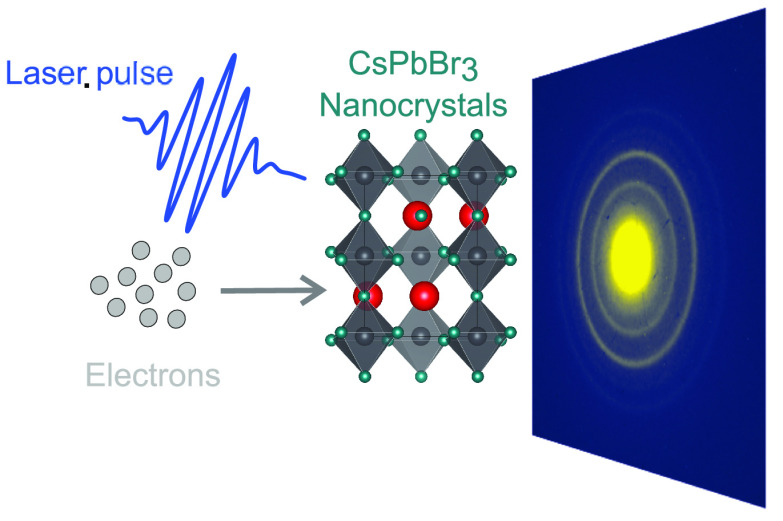

The microscopic origin of slow hot-carrier cooling in lead halide perovskites remains debated and has direct implications for applications. Slow hot-carrier cooling of several picoseconds has been attributed to either polaron formation or a hot-phonon bottleneck effect at high excited carrier densities (>10^18^ cm^–3^). These effects cannot be unambiguously disentangled with optical experiments alone. However, they can be distinguished by direct observations of ultrafast lattice dynamics, as these effects are expected to create qualitatively distinct fingerprints. To this end, we employ femtosecond electron diffraction and directly measure the sub-picosecond lattice dynamics of weakly confined CsPbBr_3_ nanocrystals following above-gap photoexcitation. While we do not observe signatures of a hot-phonon bottleneck lasting several picoseconds, the data reveal a light-induced structural distortion appearing on a time scale varying between 380 and 1200 fs depending on the excitation fluence. We attribute these dynamics to the effect of exciton–polarons on the lattice and the slower dynamics at high fluences to slower sub-picosecond hot-carrier cooling, which slows down the establishment of the exciton–polaron population. Further analysis and simulations show that the distortion is consistent with motions of the [PbBr_3_]^−^ octahedral ionic cage, and closest agreement with the data is obtained for Pb–Br bond lengthening. Our work demonstrates how direct studies of lattice dynamics on the sub-picosecond time scale can discriminate between competing scenarios proposed in the literature to explain the origin of slow hot-carrier cooling in lead halide perovskites.

Lead halide perovskites (LHPs) have attracted significant attention for their optoelectronic properties, in particular their photovoltaic performance.^1−4^ Hot-carrier cooling in LHPs occurs via several processes with time scales ranging from sub-picoseconds to microseconds. There is ongoing debate over the origin of the long hot-carrier lifetimes of several picoseconds observed in LHPs, which is of direct relevance to applications such as hot-carrier solar cells.^5^ One explanation is screening by large polaron formation, which may protect carriers from scattering by phonons and defects,^6−9^ with some studies claiming that this protection may even occur up to a microsecond time scale.^6^ At high excitation densities (>10^18^ cm^–3^), a hot-phonon bottleneck effect has also been considered to explain the observed slower hot-carrier cooling rates. In such a scenario, a strongly nonthermal population of LO phonons generated by electron–phonon coupling remains out-of-equilibrium with other phonons for several picoseconds.^10−22^ These two scenarios are expected to give rise to qualitatively different lattice dynamics, and can therefore be distinguished by such observations. Hence having direct experimental access to the lattice dynamics of LHPs can enable elucidating the microscopic origin of the slow hot-carrier dynamics in LHPs.

Time-resolved diffraction techniques are ideally suited for this task. They offer the most direct measurement of nonthermal phonon populations in photoexcited materials, therefore we expect them to be an excellent probe of hot-phonon bottleneck effects.^23−25^ Furthermore, time-resolved diffraction techniques can probe coherent as well as incoherent structural dynamics, and have recently also emerged as powerful methods to probe polaronic effects.^26−28^ Several time-resolved diffraction studies have already reported light-induced lattice dynamics of the soft lattice in LHPs.^26,29−33^ Femtosecond electron diffraction (FED) was successfully employed to monitor the formation of a rotationally disordered halide octahedral structure over several picoseconds in a MAPbI_3_ thin film^29^ and was recently applied to 2D perovskites^32^ and nanocrystals (NCs).^33^ Time-resolved X-ray diffuse scattering revealed transient strain fields building over tens of picoseconds after polaron formation.^26^ Using time-resolved X-ray absorption spectroscopy, Cannelli and co-workers were able to identify the photoinduced polaronic distortion of the lattice tens of picoseconds after photoexcitation.^31^ These works clearly demonstrate the benefits of direct structural probes of the soft LHP lattice. However, while these studies have mainly focused on processes on several picosecond time scales, investigating the sub-picosecond lattice dynamics is extremely relevant as well, as competition between hot-carrier thermalization and polaron formation is expected to occur on these time scales.

Here we employ FED to probe the sub-picosecond lattice dynamics in weakly confined CsPbBr_3_ NCs after photoexcitation above the electronic band gap. As a particular form of LHPs, NCs have drawn attention for their facile colloidal synthesis, high fluorescence quantum yield, and tunable band gap via composition and size.^2^ Perovskite NCs have been shown to host an excitonic fine structure in single NC studies.^34−36^ Many properties of NCs drastically differ from bulk ones, in particular, for NCs with sizes smaller than the exciton Bohr radius.^37^ Such small NCs essentially behave like quantum dots where physical quantum confinement gives rise to a clear exciton manifold in the room temperature linear absorption spectrum^38^ and significantly modified electronic dynamics due to enhanced Auger and multiexcitonic effects. In contrast, NCs with sizes larger than the Bohr radius are in the weak confinement regime. These large NCs cannot be considered quantum dots, and their properties were shown to follow closely the ultrafast photophysics of bulk LHPs.^16,39^ Here, the NCs we employ fall in the category of large NCs, and carrier cooling via phonons, polaron formation, and Auger processes resemble that observed in bulk-like LHPs. Therefore, we expect our findings to be relevant for LHPs more broadly.

The FED data directly reveal the emergence of a light-induced structural distortion, which builds up with a time constant ranging from 380 to 1200 fs depending on the excitation density (0.7 to 5.6 × 10^19^ cm^–3^). This observation is consistent with the establishment of an exciton–polaron population in the NCs. Throughout the paper we use the term *exciton–polaron* instead of *polaron*, as even bulk-like perovskite NCs are known to host an exciton fine structure.^34−36,40^ Combining structure factor analysis and simulations of diffraction patterns for distorted structures, we find that our data are qualitatively consistent with specific motions of the [PbBr_3_]^−^ octahedral cage, in particular, Pb–Br_2_ bond lengthening (see Figure 1a). Furthermore, all of the observables in our data are well-modeled by a similar sub-picosecond time constant. The fluence dependence of this sub-picosecond time constant can be explained by slower initial step of hot-carrier cooling at high fluences, reported in several previous studies.^11,20,41,42^ These results suggest hot electron cooling and the creation of an exciton–polaron population occur in a coupled fashion. In contrast to the clear observation of ultrafast lattice distortions in the data, no signature of a hot-phonon bottleneck effect lasting several picoseconds was observed for the investigated excitation densities. Our work demonstrates the value of measuring the lattice dynamics directly to probe the interplay of the various competing effects at the origin of long carrier lifetimes in LHPs.

**Figure 1:**
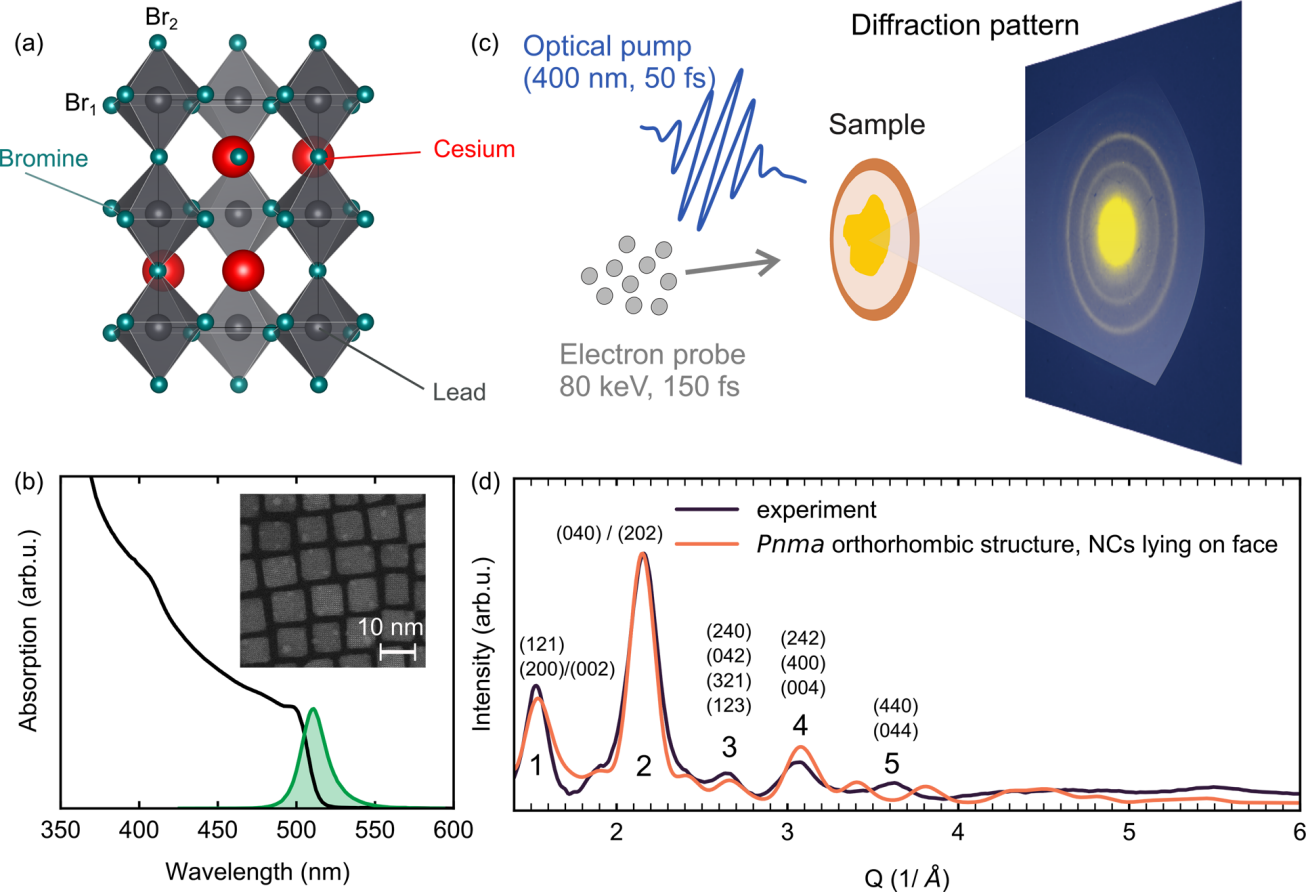
(a) Orthorhombic crystal structure of CsPbBr_3_ from ref (44), with the two inequivalent bromine atoms labeled. (b) Linear absorption (plain black line) and photoluminescence (filled green) spectra of the CsPbBr_3_ NCs dispersed in toluene. Inset: TEM picture showing the NCs in real space. (c) Schematic illustration of the FED experiment, with an example diffraction pattern of the NCs as collected by our detector. (d) Diffraction profile of the CsPbBr_3_ NCs (dark line), obtained by azimuthally averaging the pattern shown in (c). An empirical function was employed to remove background contributions. The orange curve represents the simulated pattern using the structure from ref (44) and assuming the NCs lie flat on their faces.

## Results

CsPbBr_3_ nanocrystals were synthesized following previously established procedures.^2,43^ The linear absorption spectrum of the NCs dispersed in toluene is shown as the black curve in Figure 1b, featuring a band gap of 2.5 eV (496 nm). The inset of this panel shows a representative transmission electron microscopy (TEM) image of the nanocrystals. The size of the nanocrystals is ≃10 nm, indicating weak quantum confinement effects, since the exciton Bohr radius is ≃7 nm for CsPbBr_3_.^2^ The absorption spectrum shown in Figure 1b is indeed consistent with weak confinement effects, as it does not display the excitonic progression seen for example in CdSe or CsPbBr_3_ quantum dots of much smaller sizes.^38^ The linear photoluminescence spectrum, shown as the solid green line in Figure 1b, is red-shifted by a Stokes shift of about 30 meV.

Following basic optical characterization of the samples, the NCs were drop-cast on a 10 nm thick Quantifoil TEM membrane (Plano GmbH) for the FED measurements. The NCs’ film thickness is estimated to be around 60 nm based on transmission measurements performed in an optical microscope with a narrow band-pass filter at 400 nm and previously determined values of intrinsic absorption coefficients in CsPbBr_3_ nanocrystals.^45^ An example of an equilibrium transmission electron diffraction pattern of the perovskite NCs is presented in Figure 1c. Due to averaging over a wide range of orientations of the NCs probed by the electron beam, the diffraction pattern exhibits Debye–Scherrer rings typical of polycrystalline samples. For further analysis, the diffraction pattern is azimuthally averaged and the inelastic background arising from the substrate is removed (see Supplementary Figure 1). An azimuthally averaged and background-subtracted diffraction profile is shown in Figure 1d.

The thermal equilibrium structure of perovskite NCs is characterized by a complex structural landscape, featuring local polar fluctuations among different noncubic structures,^46^ significant local distortions of the PbX_6_ octahedra,^47^ structural defects and twin boundaries.^48^ We find that the experimental pattern in Figure 1d is best reproduced by simulating the pattern for the *Pnma* orthorhombic structure, assuming that the NCs lie on one of their faces^44^ (see Supplementary Figure 2). The simulated pattern is shown as the orange curve in Figure 1d. Within the limit of the coherence length of our electron beam, the positions of the Bragg reflections in our measured diffraction pattern are consistent with the simulated pattern as well as previous experimental studies.^30,49^ The Miller indices corresponding to the peaks are labeled in Figure 1d. In the remainder of this work, we will refer to the peaks as 1–5 for convenience. The fact that NCs predominantly align on their faces significantly reduces the number of possible Miller reflections contributing at a given scattering vector in comparison with a thin film. This in turn greatly simplifies the analysis and will be key to assign the real space motions at the origin of the structural distortion.

### FED Results

FED was previously applied successfully to other types of NCs.^50−54^ A schematic illustration of the experiment is shown in Figure 1c: a femtosecond laser pulse is used to impulsively excite the electrons in the material. After a controllable time delay *t*, an electron pulse diffracts off the lattice. The resulting diffraction pattern encodes the non-equilibrium state of the lattice at *t*. By varying the time delay between the pump and the probe, the ultrafast lattice dynamics following photoexcitation can be monitored. Further details about the FED instrument are available elsewhere.^55^ Here, the CsPbBr_3_ NCs are photoexcited with a 50 fs light pulse with central photon energy *h*ν = 3.1 eV (400 nm), roughly 0.6 eV above band edge. All measurements are performed at room temperature. The incident fluence on the sample is varied in the range from 0.09 to 0.70 mJ/cm^2^, and the resulting initial density of photoexcited carriers induced by the pump pulse is estimated to be in the range from *n*_e_ = 0.7–5.6 × 10^19^ cm^–3^ (see Supporting Information). At these carrier densities, we estimate that each NCs hosts multiple excited charge carriers (see Supporting Information). After photoexcitation of the CsPbBr_3_ NCs, we follow the ensuing lattice dynamics by investigating changes in the diffraction patterns as a function of pump–probe delay.

**Figure 2:**
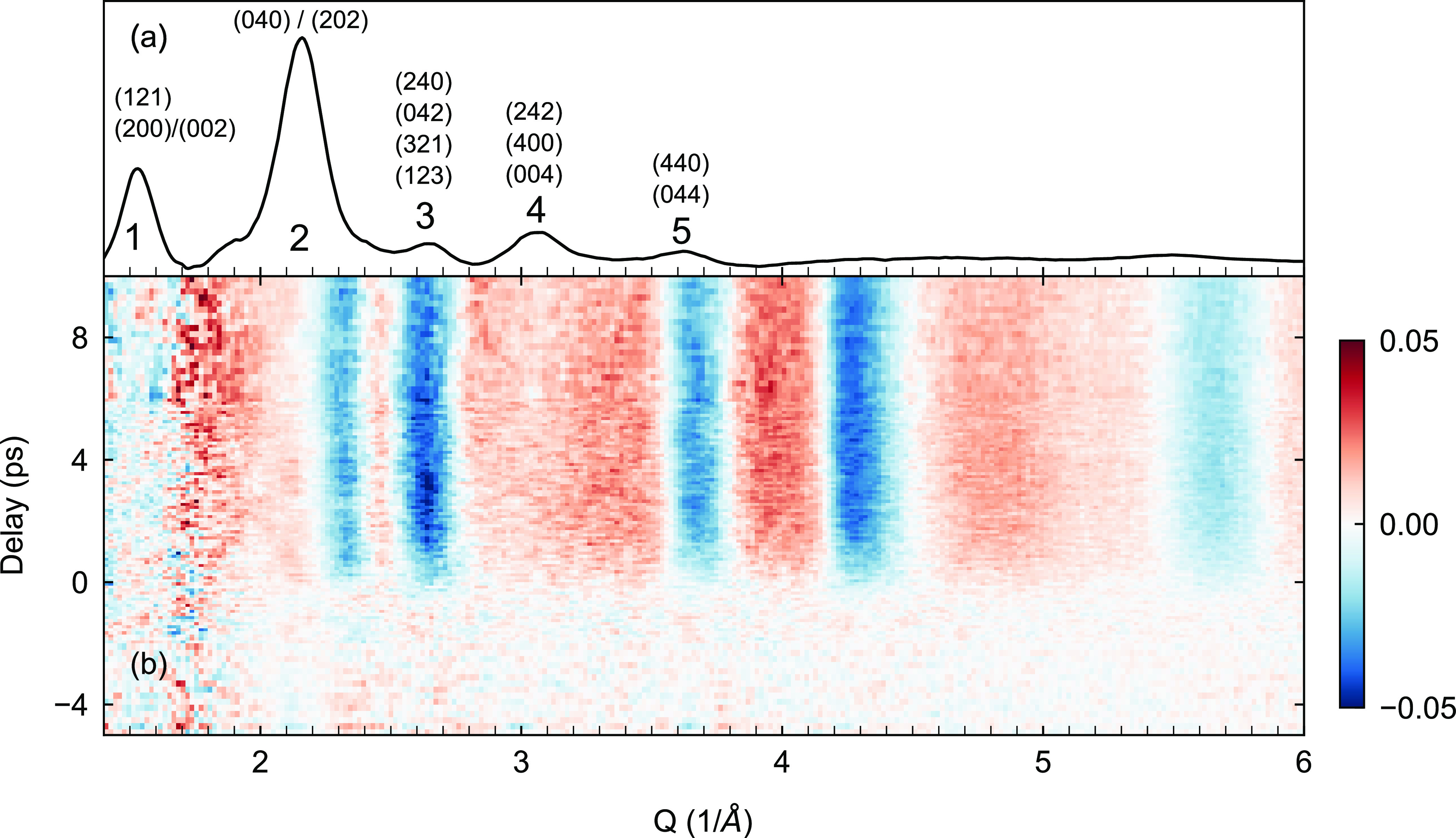
(a) Same as in Figure 1d, reproduced for convenience. (b) Relative intensity difference map shown here for an excitation density of 2.8 × 10^19^ cm^–3^.

Figure 2 presents an overview of the photoinduced lattice dynamics, in the form of relative intensity difference maps. These difference maps are obtained as [*I*(*t*) – *I*(*t* < *t*_0_)]/*I*(*t* < *t*_0_), where *I*(*t*) is the diffraction profile at time delay *t* and *t*_0_ is time zero. As shown in Supplementary Figure 3, the observed lattice dynamics remain qualitatively the same for all measured excitation densities. We verified that no time-resolved signal could be detected from the Quantifoil substrate (Supplementary Figure 4) under the same experimental conditions. In addition, the observed dynamics are reproducible over multiple scans acquired at different laboratory times (Supplementary Figure 5). The data in Figure 2 reflect complex lattice dynamics in addition to simple lattice heating. The latter was estimated to be only about 2 K for an excitation density of 2.8 × 10^19^ cm^–3^ (see Supporting Information). Thermal heating leads to an intensity decrease of all Bragg peaks as per the Debye–Waller effect; see for instance ref (56). Such a response is clearly not observed here for peaks 1, 2, and 4. Furthermore, peaks 2, 3, and 5 shift to a lower scattering vector after photoexcitation, while the position of peak 1 does not change and that of peak 4 moves to higher scattering vectors (Supplementary Figure 6). Hence the data are also inconsistent with simple thermal expansion, where all peaks would go to lower *Q* vectors. Finally, the pump-induced signals around 4.3 and 5.8 Å^–1^ reflect short-range changes in the crystal structure. In this region, we observe a discrepancy between simulated and experimental equilibrium structures. This indicates small deviations between the crystal structure of our NCs and the single crystals measured in ref (44), which renders analysis of these features challenging. The simple overview of the data in Figure 2 therefore suggests that the photoinduced lattice dynamics reflect some more complex light-induced structural distortion arising from electron–phonon interactions.

**Figure 3:**
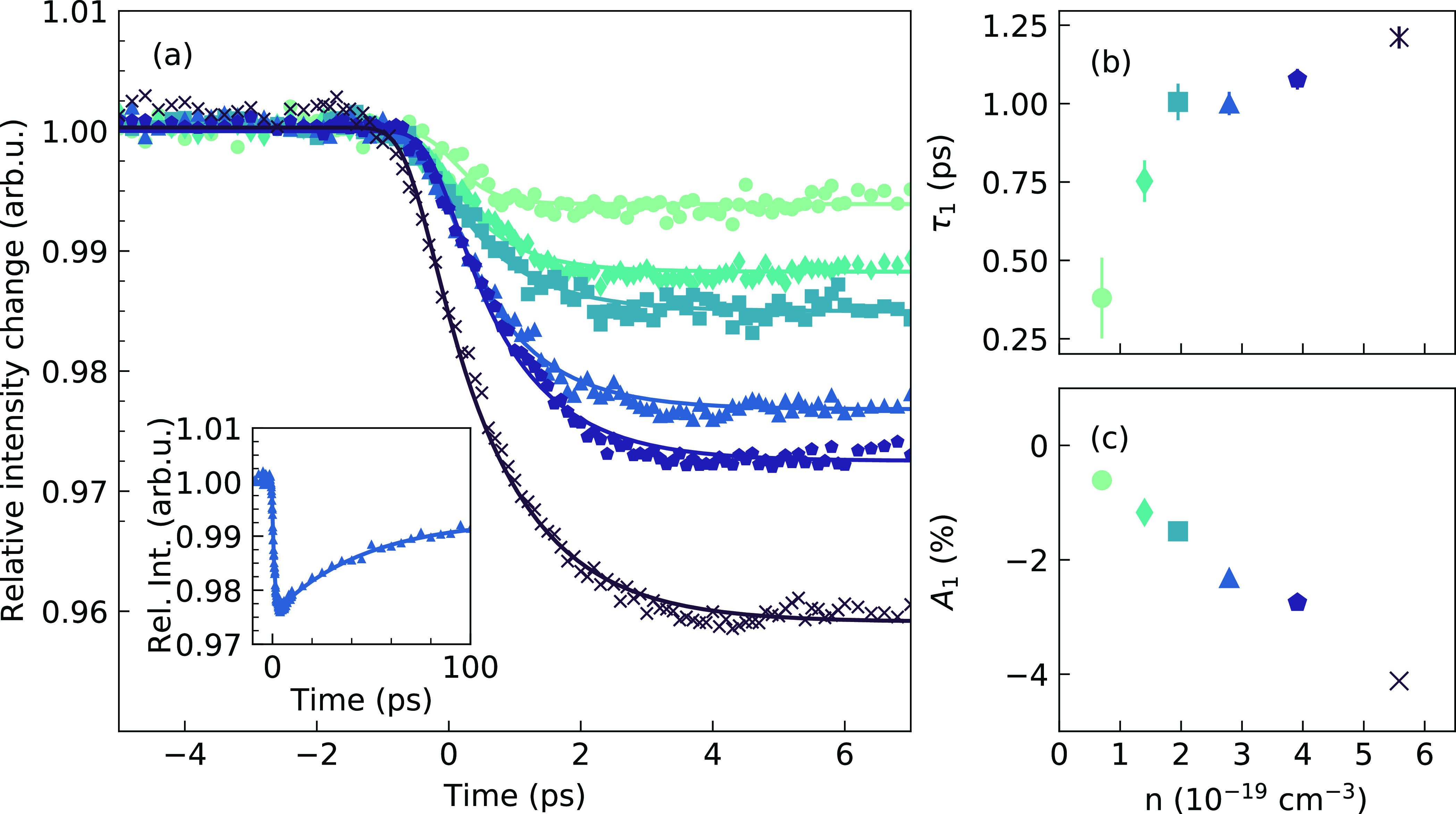
(a) Time-resolved relative diffraction intensities of the CsPbBr_3_ NCs for various excitation densities, obtained by averaging the raw diffraction signals over some regions of interest (see Supplementary Figure 7 for more details on how they were obtained). The color code is matched to that of panels (b) and (c), which display the values of the corresponding excitation densities on their *x*-axis. Inset: Example of a time-resolved trace over the 100 ps time range. Following the drop in intensity, the subsequent recovery indicates the onset of lattice cooling to the substrate. (b) Time constant τ_1_ extracted from a biexponential fit to the data as a function of excitation density. The errors correspond to 68% confidence intervals of the fits. (c) Amplitude *A*_1_ extracted from the same fit as a function of excitation density.

Figure 3 shows the time-resolved relative diffraction intensities of the CsPbBr_3_ NCs for various excitation densities, obtained by averaging the raw diffraction signals over the regions of interest (ROIs) shown in Supplementary Figure 7. The ROI approach was retained over peak intensities extracted from peak fitting, as it yielded a better signal-to-noise ratio. Furthermore, all the ROIs exhibit the same dynamic response, therefore justifying the averaging step. Indeed the main purpose of the analysis shown in Figure 3 is to extract time constants in a reliable fashion. The analysis of the structural distortion is carried out independently in the next subsection. An extended time range is presented in the inset of panel (a). The transient diffraction intensity can be fitted to a biexponential function convolved with a Gaussian (full width at half-maximum of 300 fs) to account for the finite temporal resolution of the experiment; see solid curves in Figure 3a. The fit results reveal that the lattice dynamics are well-captured by two time constants: a sub-picosecond time constant τ_1_ associated with the initial decrease in peak intensity and a slow time constant τ_2_ of around 20 ps. We assign the slow time constant to heat transfer from the NCs to the Quantifoil substrate and do not analyze it further. The fast time constant τ_1_ is intrinsic to the CsPbBr_3_ NCs and reveals the response of the lattice to the excitation. Figure 3b,c shows the evolution of τ_1_ and the associated fit amplitude *A*_1_ as a function of excitation density. We observe that τ_1_ rises with increasing excitation density, from 0.38 ± 0.13 ps at 0.7 × 10^19^ cm^–3^ to 1.17 ± 0.03 ps at 5.6 × 10^19^ cm^–3^. Meanwhile, the fit amplitude of the decay, *A*_1_, increases from about 0.5% to around 4%. This indicates, as expected, that the effect becomes more pronounced at high excitation densities.

To complement the analysis shown in Figure 3, we determine the fluence dependence of the peak position variation of peak 2 (Supplementary Figure 8), as well as the peak position dynamics for all resolvable peaks in the diffraction pattern at a chosen excitation density (Supplementary Figure 6). Here also, we fit the peak position dynamics to a biexponential function convolved with a Gaussian. In Supplementary Figure 8, we observe similar values and trend for the fast time constant, τ_1_^p2^, compared to those in Figure 3b. In contrast, no clear trend is seen for the amplitude of the peak shift, *A*_1_^p2^, as a function of fluence. As can be seen in Supplementary Figure 6c, the retrieved fast time constants of the various peaks are similar within error margin. Together, the results of Figure 3 and Supplementary Figures 6 and 8 suggest all observables in the data (relative intensities and peak positions) follow the same sub-picosecond dynamics and do not reflect independent processes. In the Supporting Information, we further show that such fast peak position changes do not violate speed of sound propagation in the specific case of NCs, owing to their high surface to volume ratio. On a general level, one can therefore conclude that the small size of any NC, whether or not physical quantum confinement plays a role, facilitates the observation of ultrafast lattice distortions.

### Analysis of the Structural Distortion

**Figure 4:**
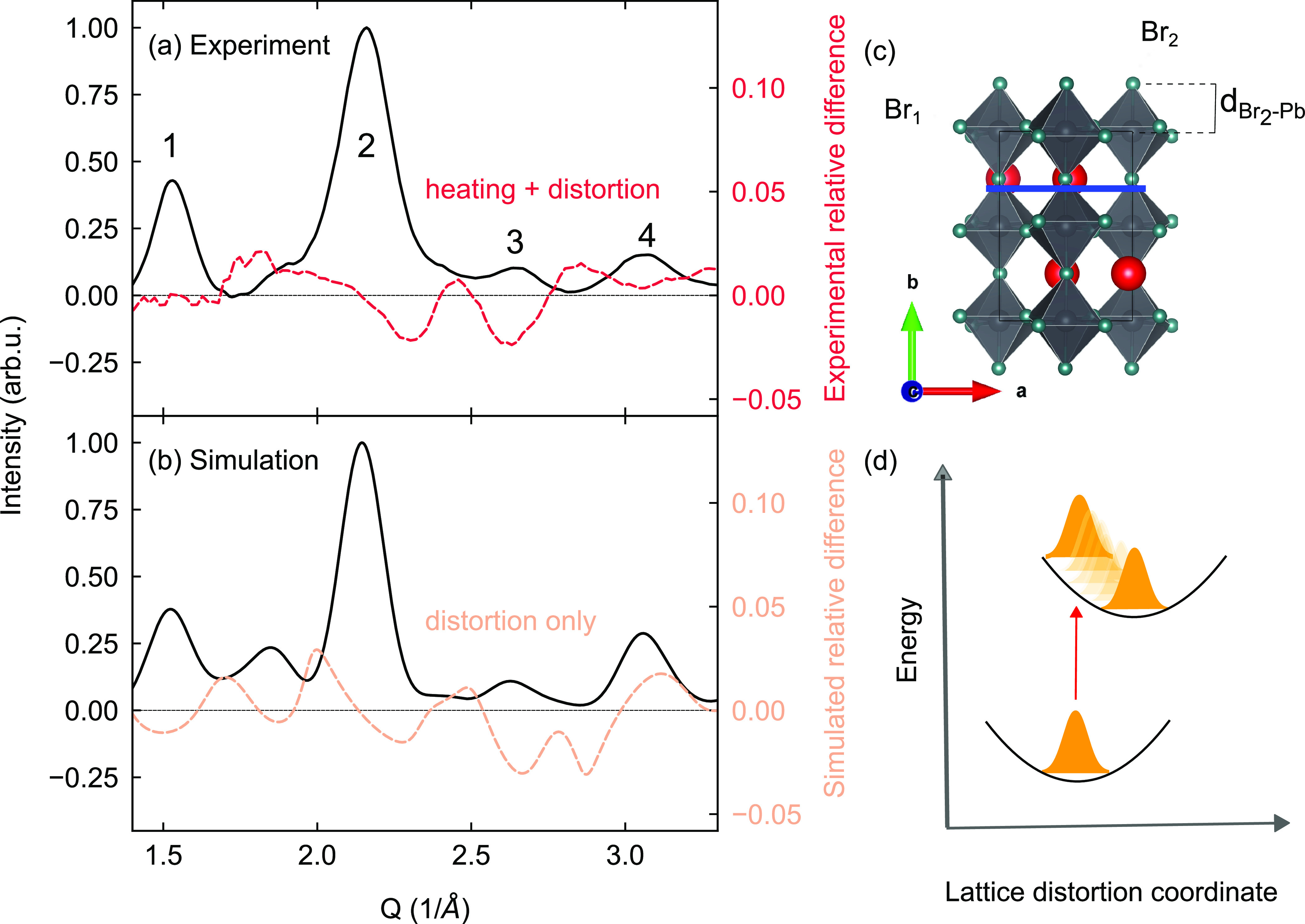
(a) Experimental diffraction profile of the CsPbBr_3_ NCs (black) and relative difference profile from the experiment (dashed red). (b) Simulated diffraction profile of the CsPbBr_3_ NCs (black) and simulated relative difference profile (dashed orange). More details about the distortion simulations are found in the text and Supporting Information. (c) The (040) Miller plane is indicated (blue). The distortion simulated in panel (b) consists of a lengthening of Pb–Br_2_ bond by 0.09%, estimated from the relative shift of peak 2. (d) Schematic illustration of the exciton–polaron formation process. The collective lattice dynamics following photoexcitation (red arrow) result in excited-state dynamics (orange wavepackets) on the excited potential energy surface that evolve from an initial state toward equilibrium.

We next evaluate possible real-space atomic motions at the origin of the structural distortion. We investigate commonly observed distortions in perovskites and whether they can give rise to the lattice dynamics in Figure 2.^57^ Specifically we consider tilting and distortions of the octahedra (e.g., changes in the Pb–Br bond length). Octahedral tilting, in particular, was reported to occur in response to ultrafast photoexcitation in other perovskites such as SrTiO_3_.^58,59^ For the analysis, we follow a similar approach as used in ref (27). We use the fact that atomic motions perpendicular to a lattice plane (*hkl*) modify the corresponding scattering intensity *I*_*hkl*_, but in-plane motions do not. We start from peak 2 because it shows the clearest signature. Peak 2 is only sensitive to the (040) and (202) Miller planes, shown in Supplementary Figure 11. Having shown that the observed peak shift cannot be reproduced by intensity distribution changes between the (040) and (202) reflections, we list the possible atomic motions contributing to the signal. For the (040) plane, for instance, either a modification of the Pb–Br_2_ bond or a tilting of the octahedra along the *c*- or *a*-axes of the crystal would change *I*_040_ (see Figure 4c). A similar reasoning can be applied to the (202) plane. The octahedra tilting angle or bond length changes can be estimated based on the shift of peak 2 at late delays (see Supporting Information). Each possible distortion is individually simulated by modifying the unit cell according to these estimates, and diffraction patterns are generated for the modified structures. This procedure enables us to directly compare the simulated and experimental difference diffraction patterns for the different cases. Examples of a few distortions and simulated patterns are shown in Supplementary Figure 11. In Supplementary Figure 12, we also simulate a phase transition from the orthorhombic to the cubic phase, previously reported in a tr-XRD study on similar CsPbBr_3_ NCs by the authors of ref (30).

The best agreement with the data is reached by a lengthening of the Pb–Br_2_ bond (see Figure 4a–c). This distortion reproduces the peak shift of peak 2, the intensity reduction in peak 3, and the intensity rise of peak 4. The magnitude of the simulated relative difference is also in agreement with the experimental relative difference. Overall, the agreement remains qualitative due to heating effects being neglected (see Supporting Information) and the sheer complexity of the LHP lattice structure. However, our work strongly suggests the involvement of Pb–Br cage motions in the buildup of the light-induced distortion and in particular changes in the Pb–Br_2_ bond.

The presence of polarons in LHPs has been claimed by multiple complementary techniques, ranging from optical^8,9,60−65^ and photoemission spectroscopies^66^ to structural probes.^26,31^ For example, previous optical spectroscopy studies of the coherent phonon response have revealed how specific phonon modes couple to the electronic excitations and participate in polaron formation.^61,67−71^ These studies are mostly restricted to zone-center coherent phonons. In contrast, our FED measurements are sensitive to the structural dynamics arising from incoherent phonon modes across the Brillouin zone and reveal the overall lattice dynamics resulting from photoexcitation, which can only be accessed via diffraction-based methods. Both the time scales and nature of the lattice dynamics observed here are consistent with the polaron formation picture.^8,64,72^ Furthermore, several studies have also suggested the involvement of [PbBr_3_]^−^ cage motions in polaron formation^8,60,69,73,74^ and atomic motion along the Pb–Br_2_ direction.^31^Figure 4d summarizes our interpretation of the data, in which lattice reorganization follows photoexcitation (red arrow); i.e., the lattice evolves from an initial state toward a new equilibrium. Even at the high excitation densities employed here, the schematic illustration reflects our finding that the dominant signature in the structural dynamics of these NCs are structural distortions, as opposed to the nonthermal phonon populations expected from the hot-phonon bottleneck scenario. At such excitation densities, each NC hosts several exciton–polarons whose radii may overlap.^75^

### Interplay between Hot-Carrier Cooling and the Creation of an Exciton–Polaron Population

In addition to the light-induced structural distortion, there are lattice heating contributions to the data arising from carrier cooling. We estimate that the Debye–Waller effect generates between 0.2 and 1% peak intensity losses depending on the scattering vector and excitation density (see Supporting Information). Thus, while heating may not dominate the lattice dynamics, it also cannot be neglected. Within our instrument response function of 300 fs, we do not observe hot-carrier cooling and the emergence of an exciton–polaron population to occur in a two-step fashion. The peak shift dynamics—which can be assumed to reflect primarily the polaronic signatures—exhibit very similar time constants compared to the integrated ROIs, where lattice heating as a result of carrier cooling should clearly play a role. Therefore, our data suggest that hot-carrier thermalization and exciton–polaron population buildup occur in a coupled fashion.

The increase of τ_1_ with increasing excitation density seen in Figure 3b and in Supplementary Figure 8b shows that the structural distortion exhibits longer time constants at higher fluences. We note that the intensity variations in our experiments reflect the population dynamics of exciton–polarons, which depend on both exciton–polaron formation and hot-carrier cooling times.^60^ Multiple studies have reported a slowing down of carrier cooling on the sub-picosecond time scale at high fluences.^11,20,41,42^ Such a trend could arise from carrier screening effects at high excitation densities, which are known to occur in polar semiconductors and would reduce the rate of phonon emission.^76^ Alternatively, from a simple two-temperature model, one would also expect an increase of the lattice heating time with increasing initial change in electronic temperature.^63,77^ Finally, the same trend would also be observed in the case of nonthermal phonon populations, which are likely present in our sample on the sub-picosecond time scale given the strong dependence of the time constant with fluence. Regardless of the origin of this dependence, the slower creation of the distortion at high fluences in our data is fully consistent with the slower hot-carrier cooling rates observed by others. Further measurements pumping the NCs at the bandedge, where cooling effects are minimized, may isolate the exciton–polaron formation time in the future.

Even at the highest fluences, our measurements do not display signatures of lattice heating over a time scale of several picoseconds. At high excitation densities (>10^18^ cm^–3^), several transient-absorption (TA) studies have reported slow components in the spectral dynamics, with time constants ranging from a few picoseconds^11,13,14,16,18^ to tens or even hundreds of picoseconds.^10,12,15^ The interpretation of these slow components is controversial and lacks a commonly accepted picture,^15−17,78^ with some studies assigning the slow dynamics to the hot-phonon bottleneck effect^10^ and other studies assigning them to Auger relaxation processes.^78^ At carrier densities >10^19^ cm^–3^, Auger processes can indeed become significant in NCs, in particular, in quantum dots. These processes can also be expected to slow down the lattice dynamics on the several picosecond time scale, since they create hot-carriers which undergo cooling via phonon emission. Given the weakly confined nature of our NCs and previous studies, however, we do not expect enhanced Auger effects in our NCs compared to bulk LHPs.^79^ For our inorganic NCs, the time-resolved diffraction data do not exhibit signatures of a long-lived hot-phonon bottleneck.

## Conclusions

Our study has revealed the sub-picosecond lattice dynamics of photoexcited CsPbBr_3_ NCs. We observed a structural distortion building-up within hundreds femtoseconds, which we assigned to the lattice signature of an emerging population of exciton–polarons. Using structure factor analysis, we showed that the distortion is consistent with atomic motions of the [PbBr_3_]^−^ cage. We further observed that the exciton–polaron population takes more time to build-up at high fluences, which we attributed to slower hot-carrier cooling. In contrast to the clear observation of structural distortions, no hot-phonon bottleneck effect lasting several picoseconds was observed for the investigated excitation densities, which nearly reached the damage threshold of the NCs. Our data thus demonstrate that the structural dynamics in these photoexcited NCs is dominated by ultrafast lattice distortions, thereby enabling us to discriminate between the competing scenarios proposed in the literature to explain the slow hot-carrier cooling in lead halide perovskites.

## Methods

### Femtosecond Electron Diffraction Experiments

Part of the output of a Ti:sapphire ultrafast amplifier (Astrella, Coherent, 800 nm, 4 kHz, 6 W, 50 fs) is used to generate 400 nm pump pulses via second harmonic generation (SHG) in a beta barium borate (BBO) crystal. Another part of the main laser beam feeds a one-stage home-built noncollinear optical parametric amplifier (NOPA) which is used to generate 500–800 nJ pulses centered around 500 nm. These pulses are sent to a prism-compressor setup for dispersion management and subsequently rooted and focused onto the gold photocathode of the electron gun. Electrons are generated via photoemission resulting from two-photon absorption on the photocathode. For all of the experiments conducted in this study, the generated electrons are accelerated toward the anode to an energy of 80 keV. The femtosecond electron bunches exit the anode through a small hole and encounter the sample after propagating for around 1 mm. They diffract off the sample and are focused by a magnetic lens onto a detector (F416, TVIPS). More details about the FED setup can be found in ref (55).

## Supporting Information Available

Simulated diffraction profiles and Bragg peaks assignment; calculation of excitation density; estimate of temperature rise; estimate of the Debye–Waller effect; estimate of average distance of atom to nanocrystal surface; determination of octahedral tilt angle (PDF)

## Notes

A preprint version of this paper can be found under Hélène Seiler; Daniela Zahn; Victoria C. A. Taylor; Maryna I. Bodnarchuk; Yoav William Windsor; Maksym V. Kovalenko; Ralph Ernstorfer. Direct observation of ultrafast lattice distortions during exciton–polaron formation in lead halide perovskite nanocrystals. 2022, 2209.05931. arXiv. arxiv.org/abs/2209.05931 (accessed January 4, 2023). The data presented here are available on a data repository.^80^
