# Supplementary material for: Direct Observation of Ultrafast Lattice Distortions during Exciton–Polaron Formation in Lead Halide Perovskite Nanocrystals
Source: ACS Nano. 2023 Jan 18;17(3):1979–88. doi: 10.1021/acsnano.2c06727 (PMC9933605; doi:10.1021/acsnano.2c06727)
Supplement: Supplementary file 1 — nn2c06727_si_001.pdf [file nn2c06727_si_001.pdf]

Supporting Information: Direct observation of ultrafast lattice distortions during exciton-polaron formation in lead halide perovskite nanocrystals

Hélène Seiler,[*,†,‡] Daniela Zahn,[†] Victoria C. A. Taylor,[†] Maryna I. Bodnarchuk,[¶] Yoav William Windsor,[†,§] Maksym V. Kovalenko,[‖,¶] and Ralph Ernstorfer[*,†,§]

†*Fritz Haber Institute of the Max Planck Society, Faradayweg 4-6, 14195 Berlin, Germany*

‡*Physics Department, Free University of Berlin, Arnimallee 14, 14195 Berlin, Germany*

¶*Laboratory for Thin Films and Photovoltaics, Swiss Federal Laboratories for Materials Science and Technology, Überlandstrasse 129, CH-8600 Dübendorf, Switzerland*

§*Institut für Optik und Atomare Physik, Technische Universität Berlin, Straße des 17. Juni 135, 10623 Berlin, Germany*

‖*Institute of Inorganic Chemistry, Department of Chemistry and Applied Biosciences, ETH Zürich, CH-8093 Zürich, Switzerland.*

E-mail: seiler@fhi-berlin.mpg.de; ernstorfer@fhi-berlin.mpg.de

Supplementary Text

Simulated diffraction profiles and Bragg peaks assignment

We simulate the diffraction peak positions and intensities of $CsPbBr_3$ with the *Crystal-Maker* software as well as Python scripts, using the crystallographic information from Ref. 1. The cell parameters are $a = 8.24$ Å, $b = 11.74$ Å and $c = 8.20$ Å (orthorhombic *pnma*). Supplementary Figure 2 shows a comparison between the experimental pattern and various simulated radial diffraction profiles. Panel (a) shows the simulated pattern assuming a completely random orientations of the NCs, akin to a powder pattern. While most simulated peak positions seem to match experimental ones, peak intensities are not well reproduced. A better agreement of both peak positions and intensities is reached assuming that the NCs preferably lie on one of their faces, but with that face at random angle with respect to the substrate. To simulate this scenario, we compute the single crystal diffraction pattern assuming the electron beam is travelling along the b (or a, c) axis of the crystal, and we rotate that pattern around its center from 0 to 2π. Then we find the linear combination of the three patterns (along the a, b and c directions) that best reproduces the experimental pattern. The results of this procedure are shown in panel (b). A much better agreement between the experimental and simulated peak positions and intensities is achieved compared to the powder pattern case. As a further point of reference, in panel (c) we also show a pattern similarly simulated but assuming a cubic structure based on the crystallographic information from Ref. 2. As can be seen from comparing panel (c) with panel (b), the measured diffraction pattern resembles more the orthorhombic phase, consistent with previous measurements at room temperature.[1] Finally, in panel (d) we report the powder pattern of $PbBr_2$, which we would expect if the sample was significantly degraded.[3] We observe a complete lack of agreement between the simulated pattern of $PbBr_2$ and the experimental pattern.

Based on the results in Supplementary Figure 2, we proceed to the assignment of peaks

1-8 to specific Miller indices assuming the orthorhombic structure. The Miller indices and diffraction intensities corresponding to peaks 1-5 are shown in tables 1-5 below. The intensities are scaled with respect to the max intensity I_{max}, obtained for the (040) reflection. We note that in the reciprocal space region spanning $4 < Q < 6$ [1/Å], corresponding to peaks 6-8, there are more than 90 Bragg reflections with I/I_{max} ranging from 2-12 %. Due to the large density of peaks, we do not proceed to an assignment of Miller indices in that region.

Calculation of excitation density

The aim is to determine the excitation densities in the perovskite NCs following laser excitation. We start by considering the photon energy of 3.1 eV (400 nm):

$$E_{\mathrm{ph}} = \frac{\mathrm{hc}}{\lambda} = \frac{2 \times 10^{-25}\,\mathrm{J} \times \mathrm{m}}{400 \times 10^{-9}\,\mathrm{m}} = 5 \times 10^{-19}\mathrm{J} \tag{1}$$

The incident number of photons per area is:

$$N_{\mathrm{ph,in}} = \frac{F_{\mathrm{i}}}{E_{\mathrm{ph}}} = \frac{0.09 \times 10^{-3}\mathrm{J/cm}^{-2}}{5 \times 10^{-19}\mathrm{J}} \approx 1.8 \times 10^{14}\,\mathrm{cm}^{-2}, \tag{2}$$

Where we take $F_i \simeq 0.09$ mJ/cm^{-2} as an example incident fluence on the sample. Next we calculate the absorbed number of photons per unit volume. The fraction of absorbed photons is A = 1 - T - R, where T and R are the transmission and reflection coefficients, respectively. We determine A from transfer matrix calculations based on the complex index of refraction of $CsPbBr_3$ at 400 nm and the estimated film thickness of 60 nm.[4,5] Hence the number of absorbed photons per unit volume is:

$$N_{\mathrm{ph,abs}} = \frac{A \times N_{\mathrm{ph,in}}}{d} = \frac{0.24 \times 1.75 \times 10^{14}\mathrm{cm}^{-2}}{60\,\mathrm{nm}} \approx 7 \times 10^{18}\,\mathrm{cm}^{-3}, \tag{3}$$

Taking into account the volume of a NC, $V_{\mathrm{NC}} = 1 \times 10^{-18}$ cm^3, then number of excitations per NC can then be roughly estimated by:

$$N = N_{\mathrm{ph,abs}} \times V_{\mathrm{NC}} = 7 \times 10^{18}\,\mathrm{cm}^{-3} \times 1 \times 10^{-18}\,\mathrm{cm}^3 \approx 7 \tag{4}$$

We see that at the high excitation densities employed in our study, we have a large number of excitations (N $\gg$ 1) per nanocrystal. Table 1 summarizes the incident fluences F_i and the resulting excitation densities.

F_i [mJ/cm^2]	0.09	0.17	0.24	0.35	0.49	0.70
$N_{\mathrm{ph,abs}}$ [$10^{19}\times$ cm^{-3}]	0.70	1.40	1.95	2.79	3.91	5.58

Estimate of temperature rise

We estimate the temperature rise in the photo-excited perovskite NCs:

$$\Delta T = \frac{E_{\rm abs}}{C} \tag{5}$$

In this expression, $C \simeq 125$ J/[mol× K] is the heat capacity of $CsPbBr_3$, from Ref. 6 and $E_{\rm abs}$ is the absorbed energy in Joule per mole. To find a value for $E_{\rm abs}$, we first calculate the excess energy per unit cell in a perovskite NC. The number of unit cells N_c in a cubic NC of 10 nm size is $N_c = V_{\rm NC}/V_{\rm uc}$, where $V_{\rm NC} = 1000\,{\rm nm}^3$ and $V_{\rm uc} = 0.85\,{\rm nm}^3$ (using the unit cell volume of the conventional standard orthorhombic unit cell in Ref. 7). This yields $N_c \simeq 1180$. In the previous section, we estimated that each NC hosts multiple excitations, each involving 600 meV excess energy with respect to the bandgap at 2.5 eV. We neglect non-radiative recombination pathways, as the photoluminescence quantum yield of these NCs was determined to be 90 %.[8] Hence, neglecting the electronic heat capacity, the absorbed energy per unit cell that ends up in the lattice is:

$$E_{\rm abs} = \frac{N \times 600}{1180} = N \times 0.51 \frac{\rm meV}{\rm unit\ cell} \tag{6}$$

Where N is the average number of excitations in a NC. Taking N = 23 (estimate corresponding to an excitation density of 2.79 × 10^{19} $\rm cm^{-3}$), we have 11.7 meV per unit cell excess energy. Each unit cell contains 12 Br atoms, 4 Cs atoms and 4 Pb atoms.[7] Hence, each of the four $CsPbBr_3$ has roughly 3 meV excess energy. We multiply this quantity by the Avogadro number to obtain the energy per mole:

$$E_{\rm abs} = 3{\times}10^{-3}{\times}6.02{\times}10^{23} = 17.60{\times}10^{20}[{\rm eV/mol}] = 1.60{\times}10^{-19}{\times}17.60{\times}10^{20}[{\rm J/mol}] \simeq 282[{\rm J/mol}]$$

Using the heat capacity of $CsPbBr_3$ of 125 J× $\rm mol^{-1}$×$\rm K^{-1}$ from Ref. 6, we can retrieve an estimated temperature rise of $\Delta T = 282/125 \simeq 2.25$ K.[9] Our estimated rise is in the

same order of magnitude as estimated in a previous FED work on $MaPbI_3$ films.[10]

Estimate of the Debye-Waller effect

We estimate the changes of the Debye-Waller (DW) factor that arise from a temperature increase ΔT, as well as its impact on relative diffraction intensities. We do so using two independent methods. In both cases the aim is to give an order of magnitude for the DW effect as opposed to a quantitative value, which goes beyond the scope of this work. In the first method, we use experimentally determined values of the atomic mean-square displacement (MSD) parameters reported in Ref. 11. From this study, the equivalent isotropic displacement parameters at 293 K for the Cs, Pb and Br atoms are $\mathrm{U_{Cs}} = 0.084$ Å^2, $\mathrm{U_{Pb}} = 0.026$ Å^2, $\mathrm{U_{Br_1}} = 0.086$ Å^2 and $\mathrm{U_{Br_2}} = 0.071$ Å^2, respectively. The average displacement is then given by $\mathrm{U_{av}} = 3/5\times(\mathrm{U_{Cs}} + \mathrm{U_{Pb}} + \mathrm{U_{Br_1}} + 2\times U_{\mathrm{Br_2}}) = 0.2$ Å^2, where the factor 3 accounts for the three independent spatial coordinates along which the atoms can vibrate. In the high-temperature limit, which is a reasonable assumption at room temperature, the MSD is proportional to the temperature. Hence we can estimate the MSD difference arising from a rise in temperature ΔT as:

$$\Delta\langle u\rangle^2 = \langle u\rangle^2(\mathrm{T} = 295\ K + \Delta\mathrm{T}) - \langle u\rangle^2(\mathrm{T} = 295\ K) = \frac{\Delta\mathrm{T}}{293\ K} \times U_{\mathrm{av}}. \tag{7}$$

Where $\langle u\rangle^2$ is the MSD. For $\Delta\mathrm{T} = 2.25$ K, we get $\Delta\langle u\rangle^2 \approx 1.6\times10^{-3}$ Å^2. We now determine how much this MSD change impacts the relative Bragg peak intensities. For the (040) reflection (peak 2), we find that $I_{040}(\mathrm{T} = 297.25\ \mathrm{K})/I_{040}(\mathrm{T} = 295\ \mathrm{K}) = e^{-\frac{1}{3}\Delta\langle u\rangle^2|\vec{G}_{040}|^2} \approx 0.9976$, with $|\vec{G}_{040}| \approx 2.14\times10^{10}\,\mathrm{m}^{-1}$. For the (044) reflection (peak 5), we find that $I_{044}(\mathrm{T} = 297.25\ \mathrm{K})/I_{044}(\mathrm{T} = 295\ \mathrm{K}) = e^{-\frac{1}{3}\Delta\langle u\rangle^2|\vec{G}_{044}|^2} \approx 0.9928$, with $|\vec{G}_{044}| \approx 3.74\times10^{10}\,\mathrm{m}^{-1}$. This means that heating effects are expected to have a non-negligible but also not dominating contribution to the observed structural dynamics.

For the second estimate of the DW factor, we consider a simple Debye model with a Debye temperature of 102 K, as obtained from literature for $\mathrm{CsPbBr_3}$.[6] We calculate $\langle u^2\rangle$ in

thermal equilibrium at 300 K using the expression from Ref. 12:

$$\langle u^2 \rangle = \frac{3\hbar}{2m} \int_0^{\omega_D} \coth \frac{\hbar\omega}{2k_\mathrm{B}T} \frac{g(\omega)}{\omega} d\omega \,, \tag{8}$$

with $g(\omega)$ the density of phonon states from the Debye model, ω_D is the Debye frequency, k_B is the Boltzmann constant and m is the average atomic mass of $CsPbBr_3$. We obtain $\langle u^2 \rangle_{300\,\mathrm{K}} \approx 0.11$ Å^2 or $\sqrt{\langle u^2 \rangle} \approx 0.33$ Å. The MSD change for $\Delta T \approx 2.25$ K retrieved is:

$$\Delta\langle u \rangle^2 = \langle u \rangle^2(\mathrm{T} = 297.25\mathrm{K}) - \langle u \rangle^2(\mathrm{T} = 295\mathrm{K}) \approx 9 \times 10^{-4}\text{Å}^2. \tag{9}$$

With this method, we find that $I_{040}(\mathrm{T} = 297.25\ \mathrm{K})/I_{040}(\mathrm{T} = 295\ \mathrm{K}) \approx 0.9986$. For the (044) reflection (peak 5), we find that $I_{044}(\mathrm{T} = 297.25\ \mathrm{K})/I_{044}(\mathrm{T} = 295\ \mathrm{K}) \approx 0.9958$. This estimates yields smaller heating effects compared to the estimated based on the U-matrix formalism. Nevertheless, they confirm that heating effects are expected to play a non-negligible role, albeit not a dominant one, in the observed structural dynamics.

Estimate of average distance of atom to nanocrystal surface

Assuming a cubic NC with an edge length of 10 nm and a unit cell length of 0.587 nm (the cubic crystal structure is considered here for the sake of simplicity), there are around 17 unit cells per edge. In total, the number of unit cells in the NC is given by $17^3 = 4913$. We now build a NC layer by layer, starting from the center unit cell. For example, the layer surrounding the center unit cell has an edge length of $N = 3$ unit cells, see Supplementary Figure 9. The number of unit cells for this layer, $N_l(3)$, can be easily shown to be 26. The counting is graphically illustrated in Supplementary Figure 9 as cubes of different colours. Based on this example, a similar counting procedure can be applied for the next layers, i.e. $N = 5, 7, \ldots$. One can easily show that the number of unit cells in a layer with an edge containing $N \geq 3$ unit cells is given by $N_l(N) = 2 \times N \times N \times +2 \times N \times (N-2) + 2 \times (N-2) \times (N-2)$. We verify that the sum of the unit cells over all the layers in the NC gives the right number of unit cells:

$$N_c = 1 + \sum_{N=3}^{17} N_l(N) = 4913 \tag{10}$$

With N in the sum an odd number. By taking the ratio $N_l(N)/N_c$, one obtains the ratio of unit cells in the NCs belonging to a given layer. For instance, the ratio of unit cells at the surface is $N_l(17)/N_c = 0.31$.

Thanks to the ratios of unit cells in each layer, we determine the average distance of an atom in the NC to the surface to be 0.97 nm. Taking the speed of sound in $CsPbBr_3$ as $v_s = 1361$ m/s,[13] the average time it would take for sound to propagate to the surface is given by $0.97 \times 10^{-9}/1361 \simeq 7 \times 10^{-13}$ s. The average time of roughly 700 fs is on the same order of magnitude as the timescale observed in the experiments. This calculation provides us with an independent estimate that it is indeed possible to observe unit cell changes in the NCs over such fast timescale. This is due to the high surface to volume ratio of nanoparticles.

Determination of octahedral tilt angle

The aim is to estimate the tilt angle α as a function of l, both indicated in Supplementary Figure 10(a). Here we consider the case of a tilt along the b-axis as an example. We assume that the Br-Br-Br angles are 90°. By definition we have $l/2 = \sqrt{2}\mathrm{b}_r$, where $\mathrm{b}_r = 4.15\times10^{-10}$ m. We can write:

$$\frac{l(\alpha)}{2\sqrt{2}\mathrm{b}_r} = \cos\beta \tag{11}$$

Since $\beta = (\pi/2 - \alpha) - \pi/4 = \pi/4 - \alpha$, we obtain:

$$\alpha(l) = \pi/4 - \mathrm{acos}\left[\frac{l}{2\sqrt{2}\mathrm{b}_r}\right] \tag{12}$$

Here l can be estimated from the shift of peak 2. A shift of 0.1 %, for example, yields $l = l_{\mathrm{ortho}} \times (1 + 0.001)$, where l_{ortho} is the value of l for the orthorhombic structure. In our case we find $\alpha \simeq 0.5°$. Supplementary Figure 10(b) shows α as a function of l/l_{ortho}.

Supplementary Figures

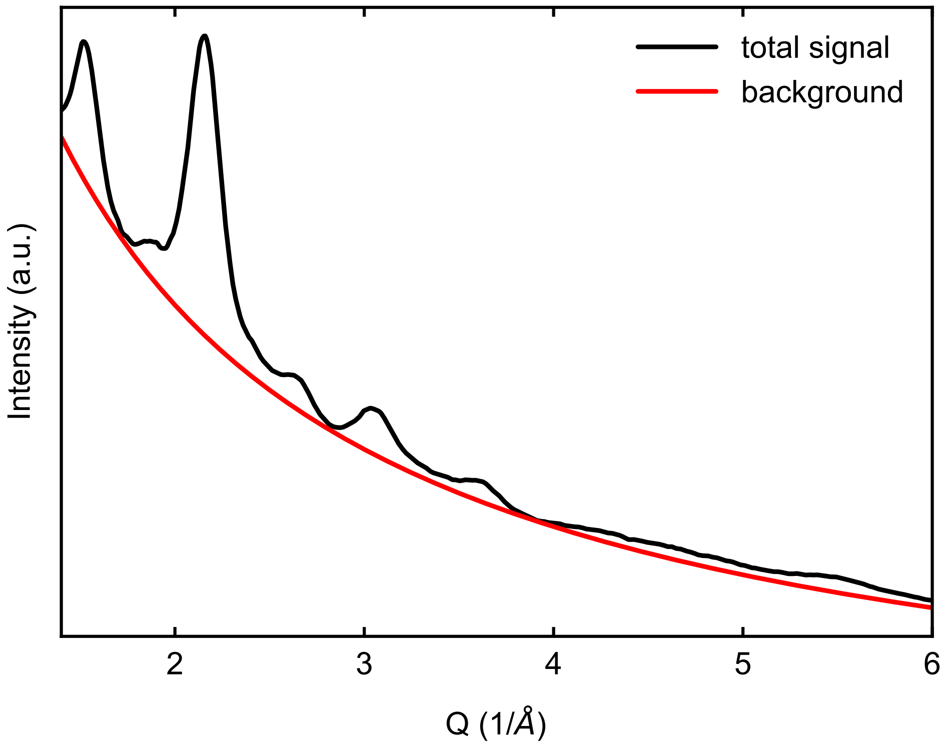

Supplementary Figure 1: Removal of background signals (red curve) arising from the substrate, diffuse scattering and contributions from the 0th order beam. Here, a Lorentzian tail and a constant offset were used as background function. We note that the qualitative features of Figure 2 in the main text remain the same whether the background-removed signals are employed or not.

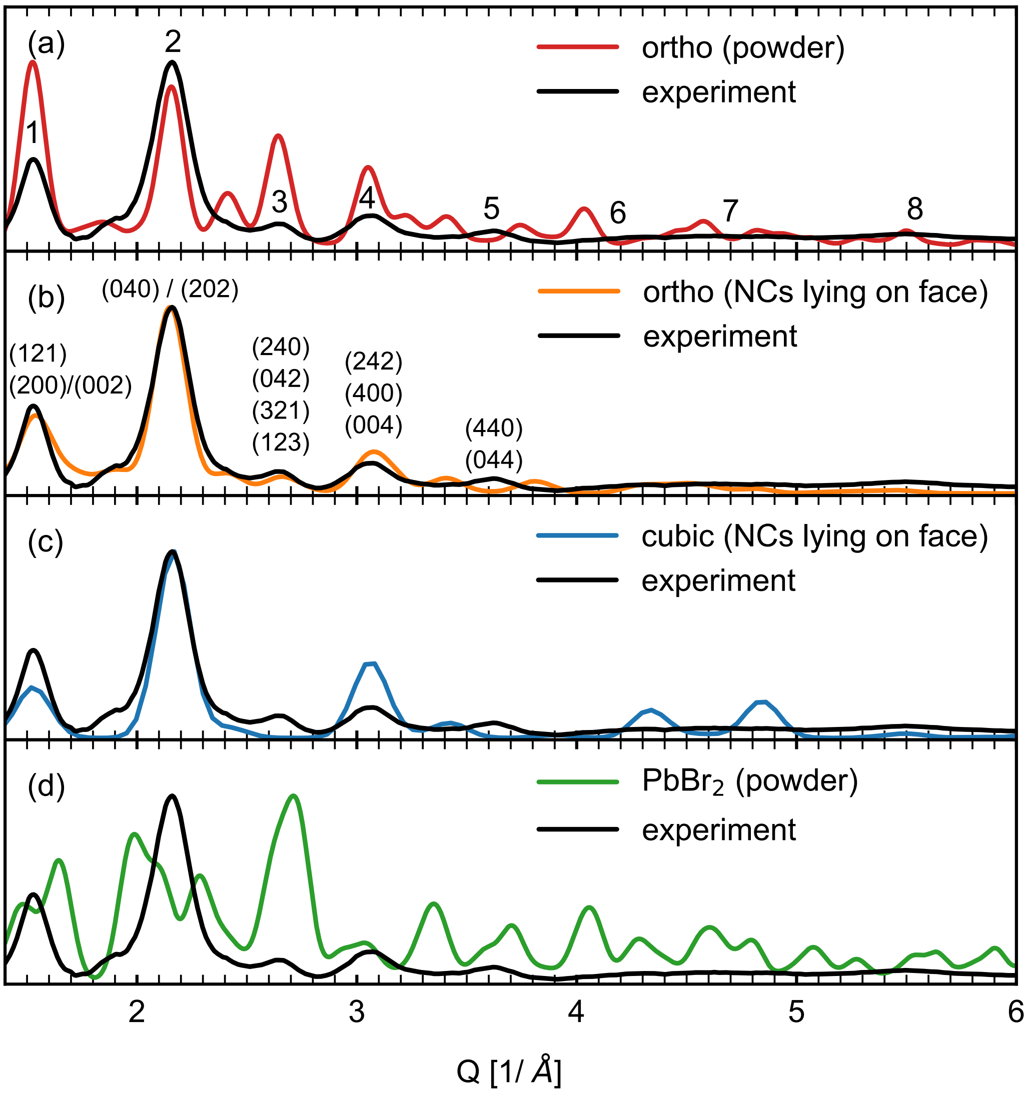

Supplementary Figure 2: Comparison of the experimental diffraction signal with various simulated patterns. (a) Simulated pattern assuming a powder-like distribution of NCs and an orthorhombic structure. (b) Simulated pattern for an orthorhombic structure, assuming the NCs lie on one of their faces on the substrate, but at random angles within the planes of the faces. More details are provided in the supplementary text. (c) Simulated pattern assuming a cubic structure, with the NCs lying on one of their faces on the substrate. (d) Simulated pattern of a $PbBr_2$ powder, expected for a significantly degraded sample.

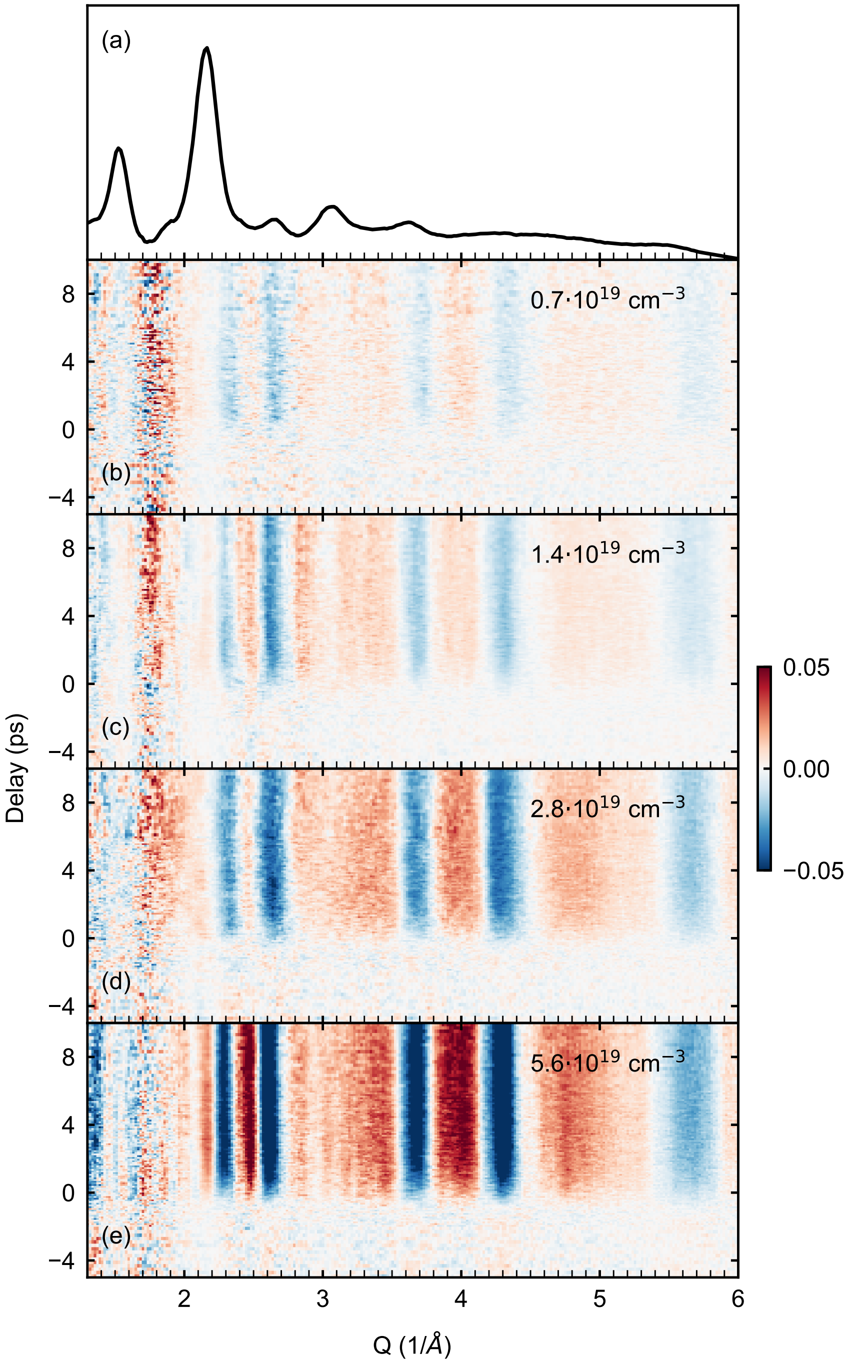

Supplementary Figure 3: (a) Background-substracted radial diffraction profile of the $CsPbBr_3$ NCs (b-e) Relative difference maps for various fluences.

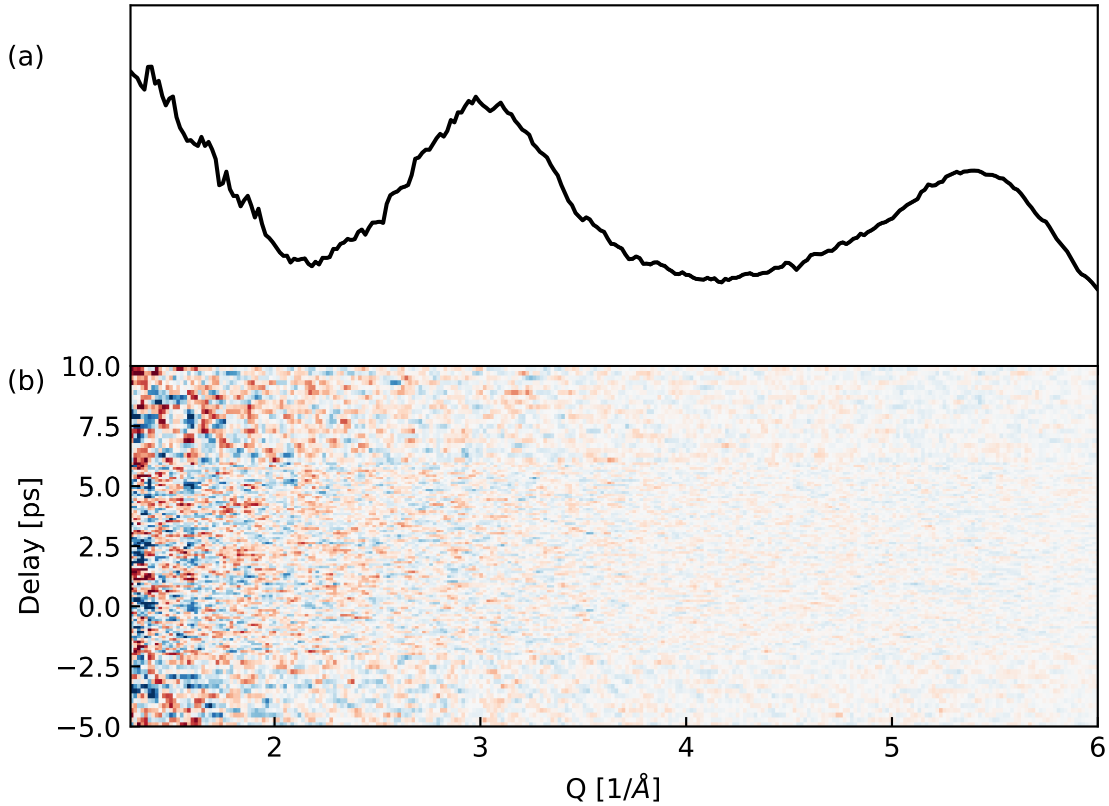

Supplementary Figure 4: Bare quantifoil (QF) response to photo-excitation at 3.1 eV with an incident fluence of 0.35 mJ/cm^2. (a) Azimuthally averaged diffraction signal of the QF membrane. Broad features can be seen around 3.0 and 5.4 [1/Å]. (b) Difference map showing no detectable photo-induced response of the QF film.

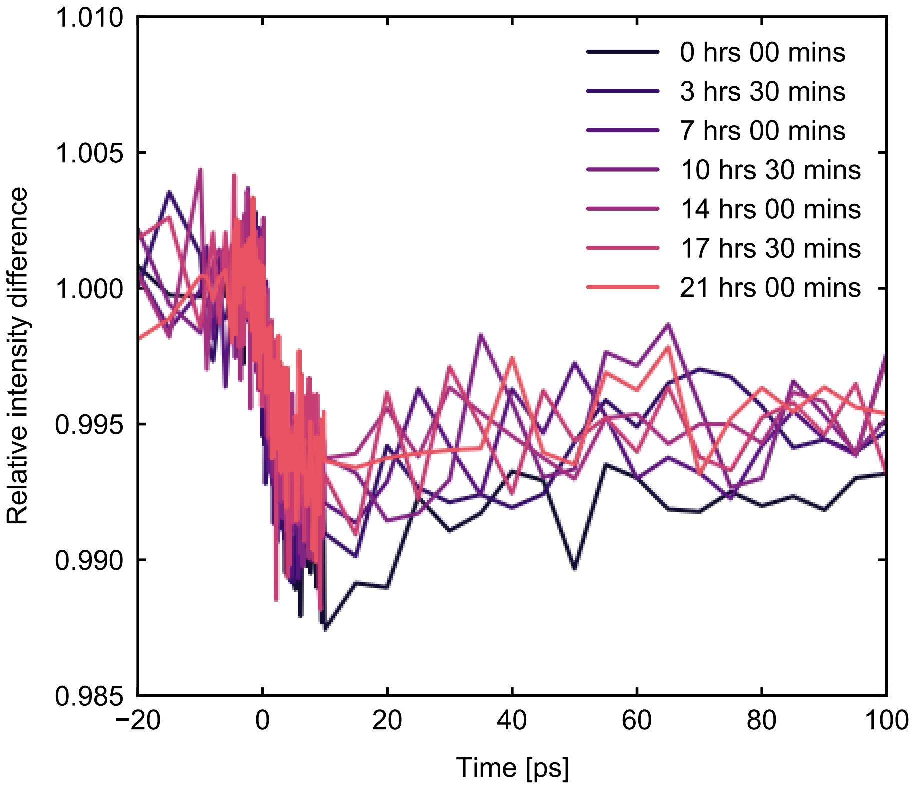

Supplementary Figure 5: Relative intensity changes of the diffraction signal integrated between Q = 3.50 and 3.77 Å (peak 5), for different repetitions of the same pump-probe delays. The caption indicates the lab time corresponding to each scan.

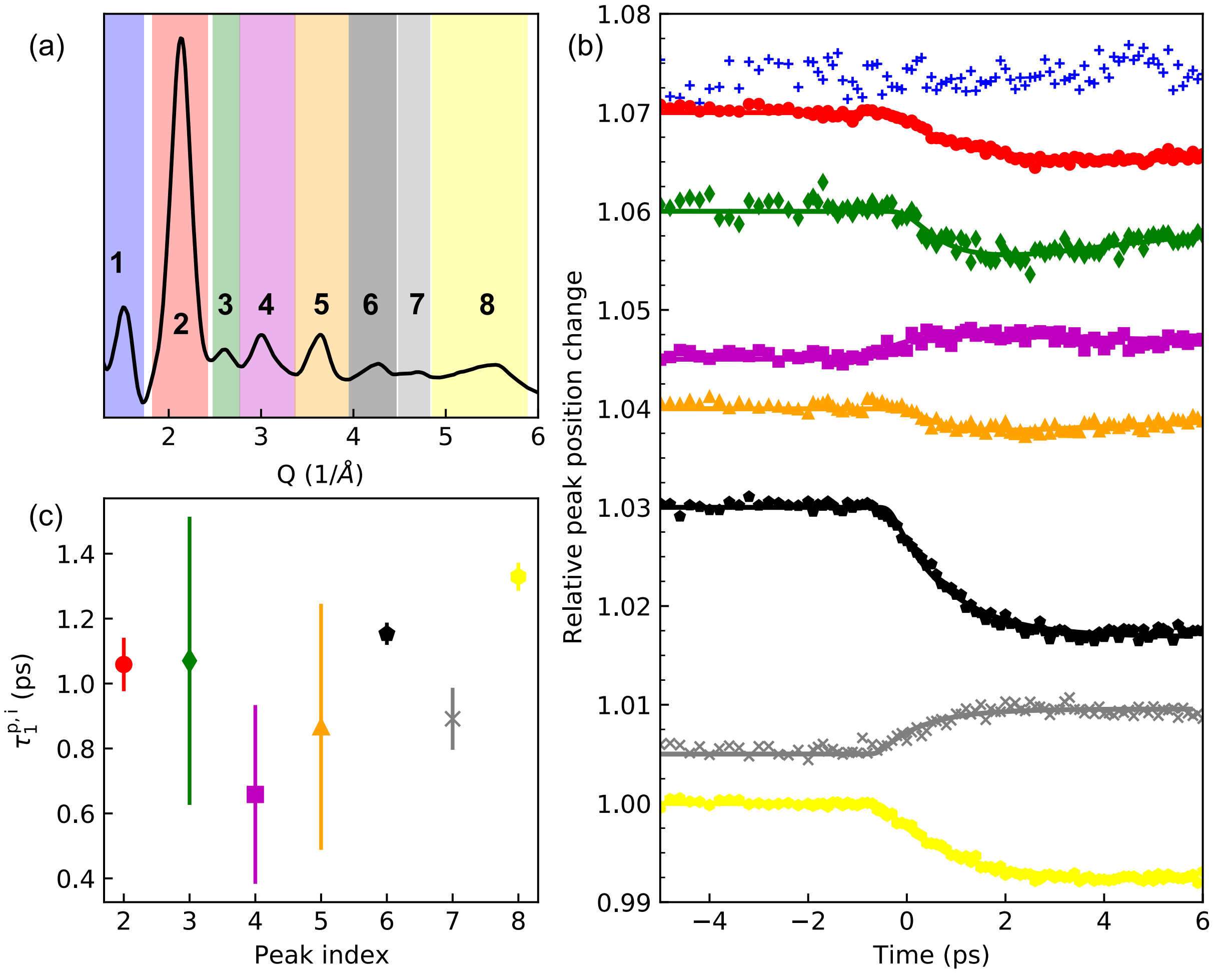

Supplementary Figure 6: Peak positions were determined as a function of time using the center-of-mass (COM) method, for the examplary excitation density of $3.91{\cdot}10^{19} \times \mathrm{cm}^{-3}$. (a) Radial diffraction profile obtained for that excitation density. Features are labeled 1-8, and the regions of interest considered to compute the COM are indicated with the colored rectangles. (b) Relative peak position change obtained via COM changes for the regions 1-8, with matching colors to those in panel (a). (c) Fast time constants $\tau_1^{p,i}$ as a function of peak index i, extracted from a bi-exponential fit to the data in panel (b).

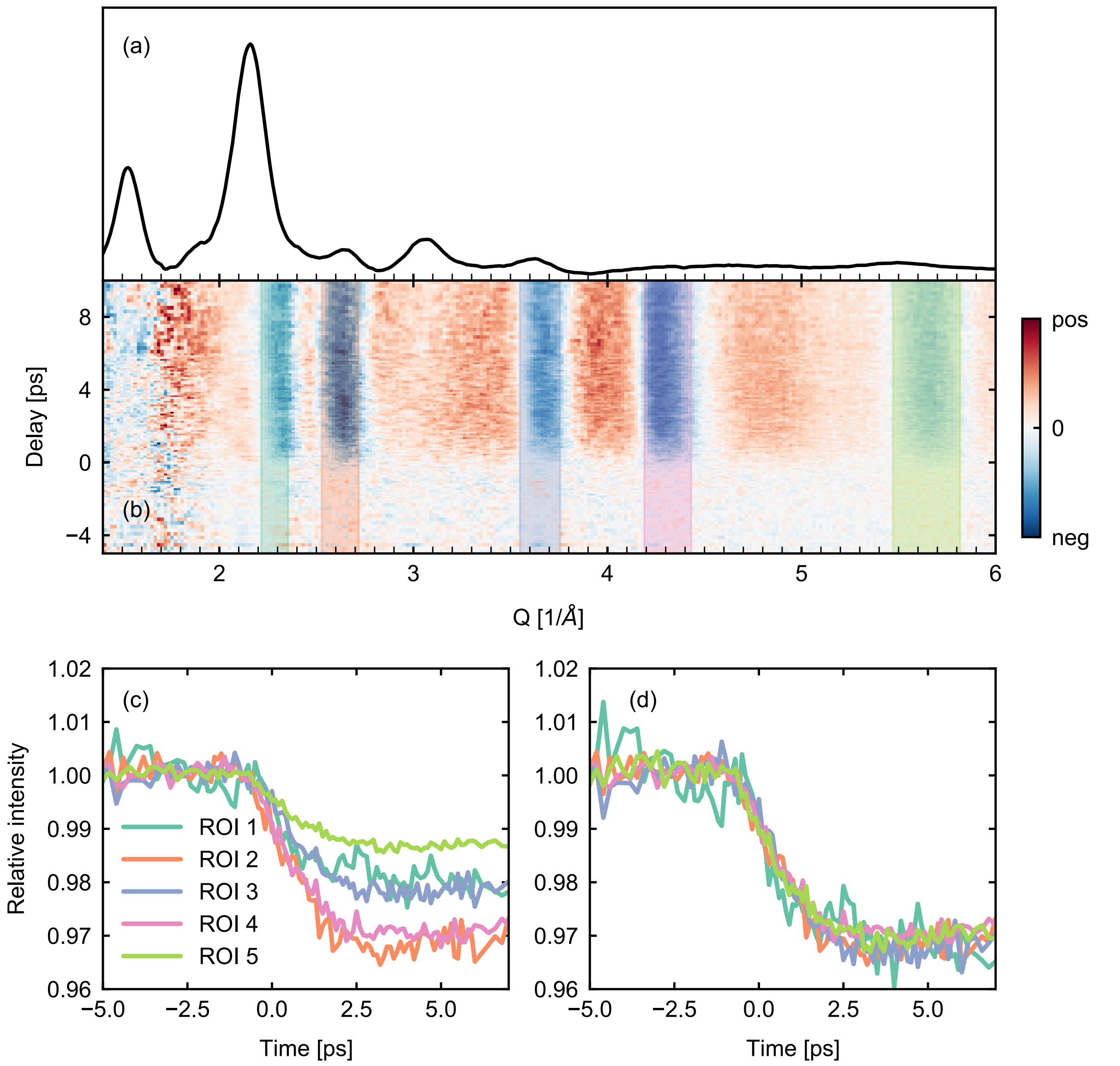

Supplementary Figure 7: (a-b) Each time-resolved trace shown in Figure 3 of the main text was obtained from the raw signals in the regions of interest (ROIs) marked by colored rectangles on the difference map. (c) This panel shows the average signal within each ROI, with matching colors to the rectangles in (b). (d) This panel shows the signals of panel (c) renormalized to the signal in ROI 2 (the largest signal). Since the signals between different ROIs are characterized by the same time constants within error margin, the average over the five ROIs was performed, yielding one time-resolved trace per excitation density. This trace is reported in Figure 3 of the main text.

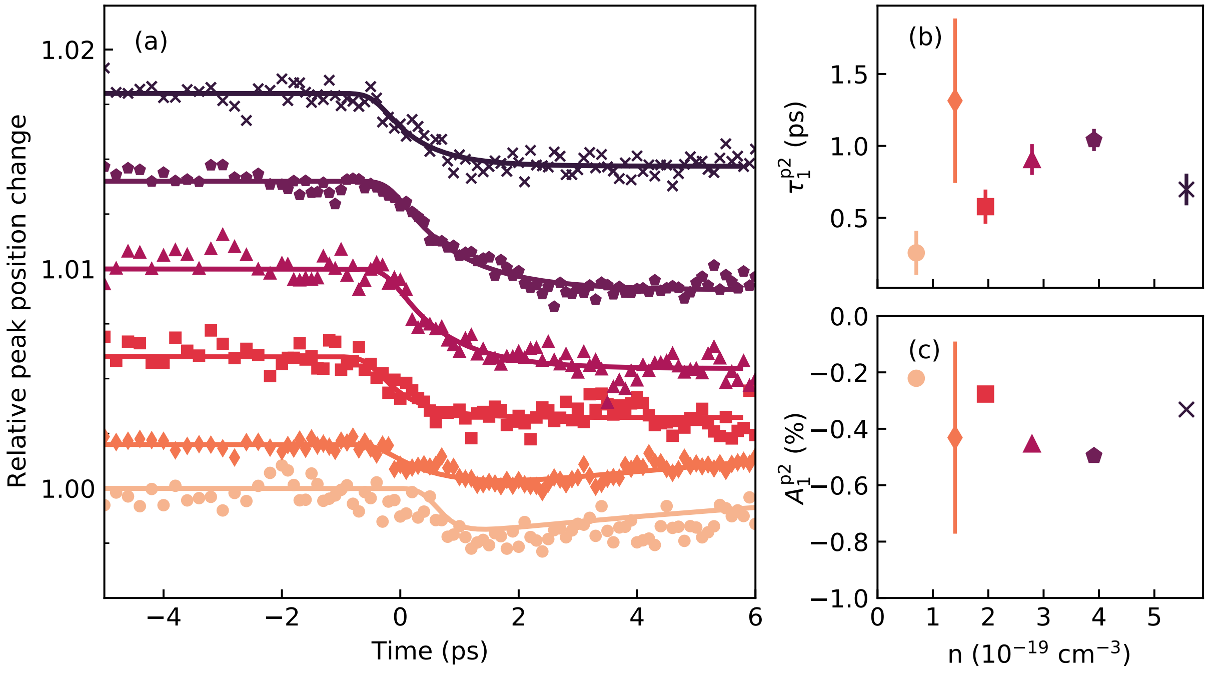

Supplementary Figure 8: (a) Relative peak position change of peak 2 as a function of pump-probe delay. The peak position changes were determined from changes in the peak center-of-mass, as this yielded the most reliable results. The color code is matched to that of panels (b) and (c), which display the values of the corresponding excitation densities on their x-axis. (b) Time constant τ_1^{p2} extracted from a bi-exponential fit to the data in panel (a) as a function of excitation density. (c) Amplitude A_1^{p2} extracted from the same fit as a function of excitation density.

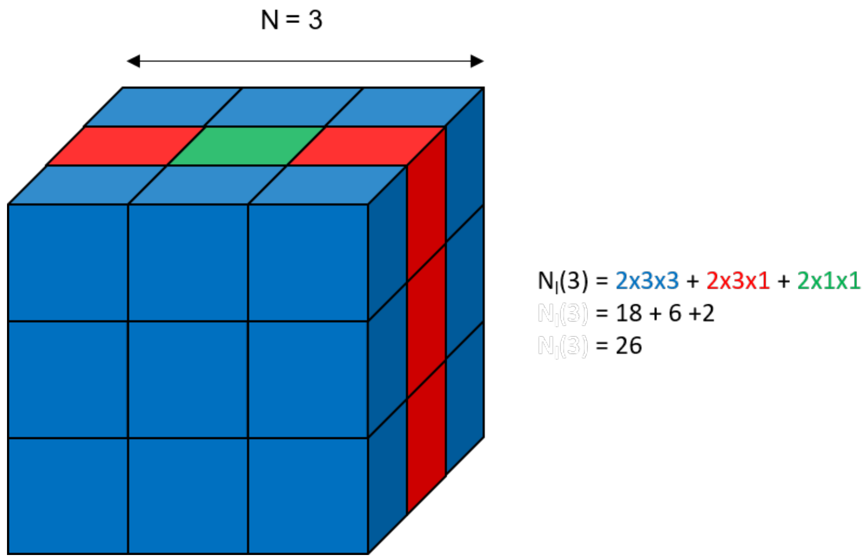

Supplementary Figure 9: Graphic illustration of how to determine how many unit cells, each represented by a cube, are contained in a given layer of the NC. Here illustrated for the layer surrounding the center unit cell of the NC, with an edge length of $N = 3$ unit cells. The number of unit cells in this layer, $N_l(3)$, is here 26.

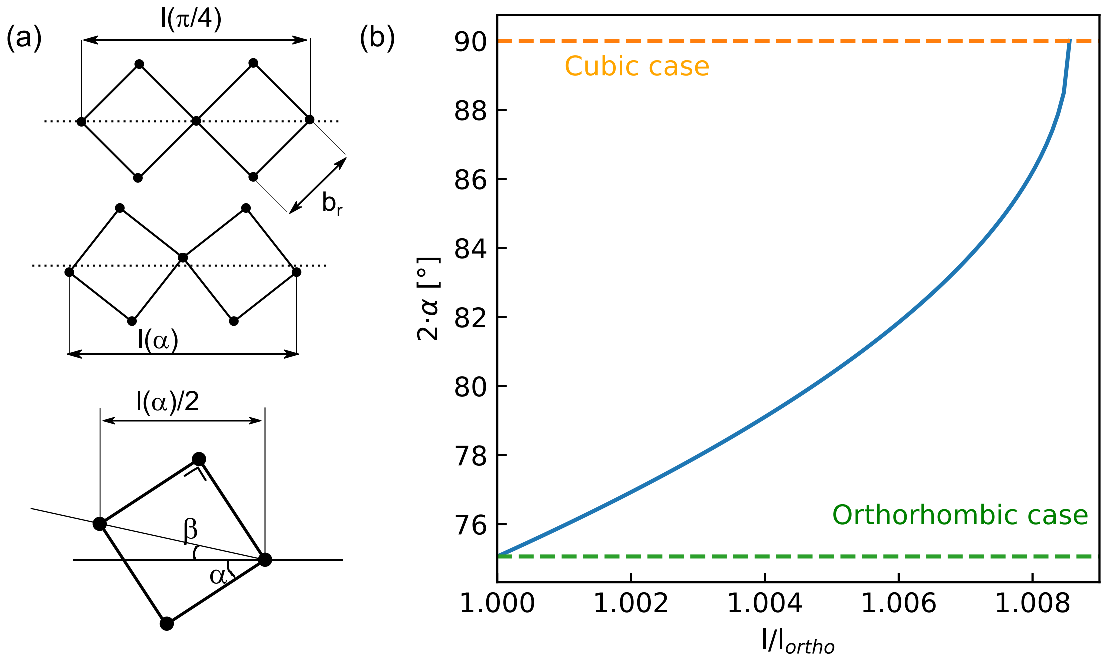

Supplementary Figure 10: (a) Octahedra from the perovskite lattice seen along the b-axis of the crystal. Only Br atoms are shown, as the black circles. The top scheme represents the cubic situation, while the middle scheme represents a case between cubic and orthorhombic. The bottom scheme defines the angles of interest. (b) Tilt angle as a function of l/l_{ortho}. We used this graph to determine the tilt angle corresponding to the shift of peak 2, which was translated in an increase of l.

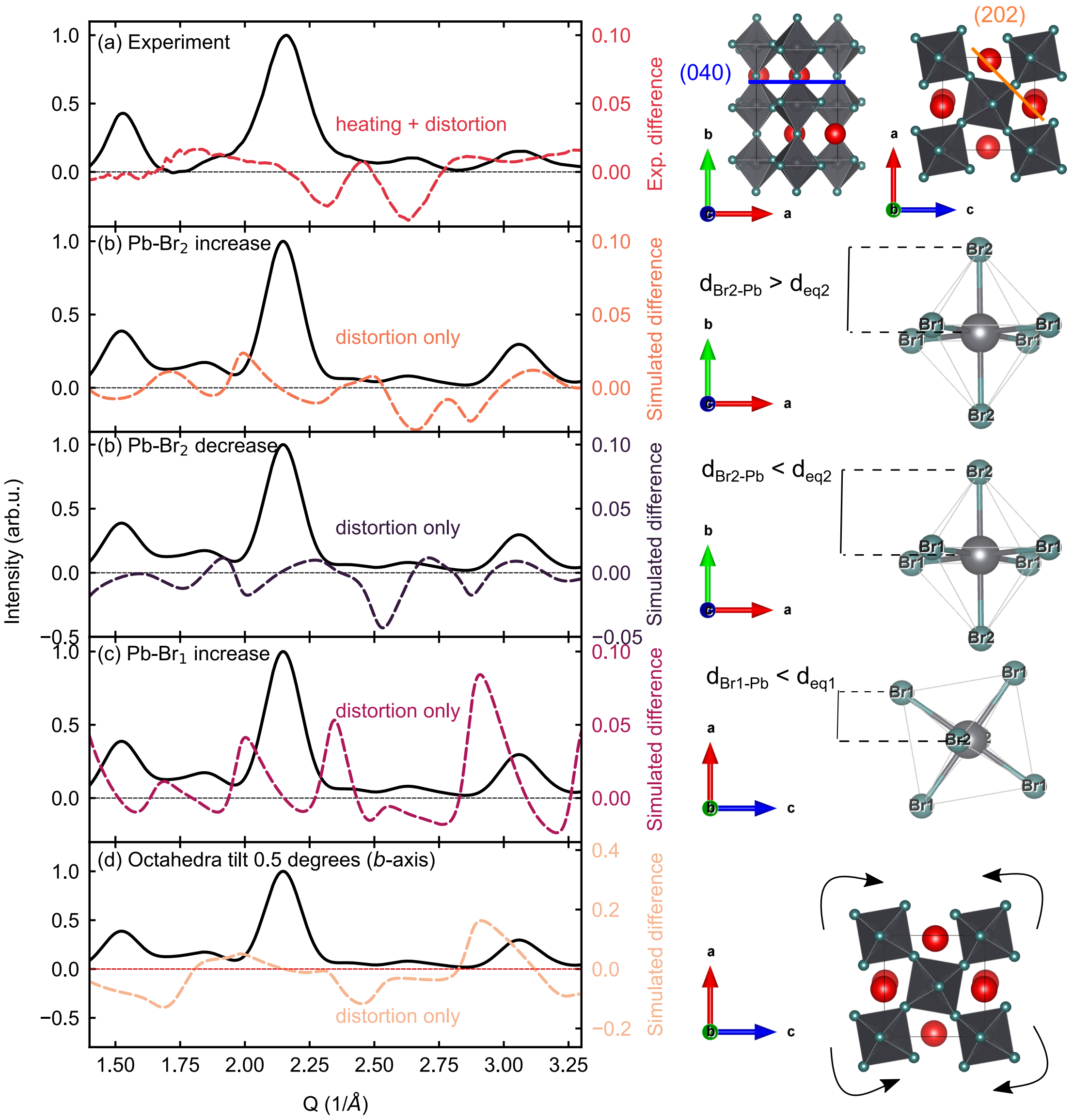

Supplementary Figure 11: (a) Experimental radial average (black) and relative intensity difference (dashed red) profiles. The difference signal is integrated over the late delays. The (040) and (202) Miller planes, to which peak 2 is sensitive, are shown on the right. (b-e) Exemplary simulated relative difference profiles (dashed lines) for various distortions. The corresponding distortion is shown on the right of each panel. Heating contributions, which would yield a negative contribution in the peak regions, are not included.

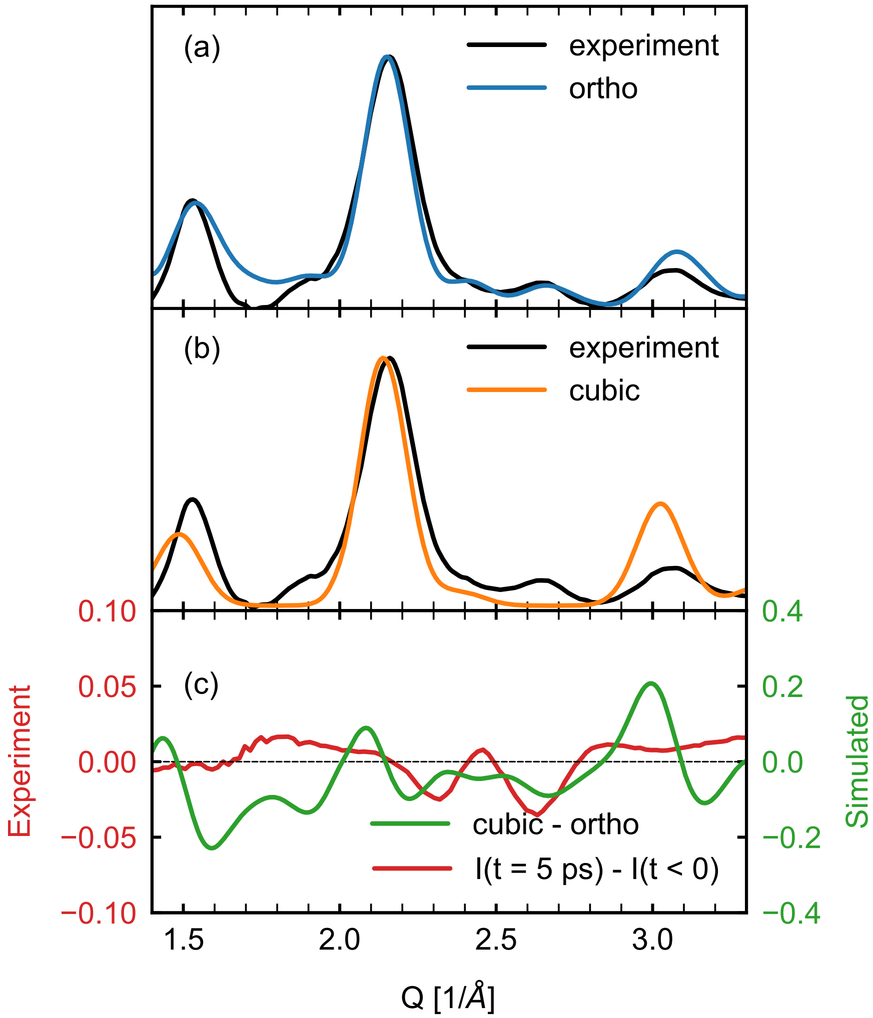

Supplementary Figure 12: Simulation of an orthorhombic to cubic phase transition in the $CsPbBr_3$ NCs. (a) Overlay of the simulated orthorhombic pattern and the experimental pattern. (b) Overlay of the simulated cubic pattern and the experimental pattern. (c) Pump-induced structural dynamics from the experiment (red) and difference between the cubic and orthorhombic patterns, simulating the fingerprints of a potential photo-induced phase transition.

Supplementary Tables

Table 1: Miller indices corresponding to peak 1 ($1.35 < Q < 1.75$ [1/Å]), as labeled in Figure 2(a) of the main text.[1] Peaks are shown with increasing value of Q. Reflections below 2 % of I_{max} are not reported. The reflections highlighted in bold constitute the vast majority of the signal measured in peak 1.

Indices	Q [1/Å]	I/I_{max} [%]
**($1\bar{2}\bar{1}$)**	**1.52**	**23.1**
**($\bar{1}2\bar{1}$)**	**1.52**	**22.2**
**($12\bar{1}$)**	**1.52**	**23.1**
**(121)**	**1.52**	**22.1**
**(200)**	**1.52**	**26.6**
**(002)**	**1.53**	**26.5**
(102)	1.71	4.3
($\bar{1}02$)	1.71	3.7
($20\bar{1}$)	1.71	3.5
(201)	1.71	4.0

Table 2: Miller indices corresponding to peak 2 ($1.95 < Q < 2.40$ [1/Å]), as labeled in Figure 2(a) of the main text. Peaks are shown with increasing value of Q. Reflections below 2 % of I_{max} are not reported. The reflections highlighted in bold constitute the vast majority of the signal measured in peak 2.

Indices	Q [1/Å]	I/I_{max} [%]
$(2\bar{2}\bar{1})$	2.01	2.9
(221)	2.01	3.2
$(22\bar{1})$	2.01	2.8
$(\bar{2}2\bar{1})$	2.01	3.3
$(12\bar{2})$	2.02	2.7
$(\bar{1}22)$	2.02	2.8
$(1\bar{2}2)$	2.02	3.1
(122)	2.02	3.2
**(040)**	**2.14**	**100.0**
**$(\bar{2}02)$**	**2.16**	**82.6**
**(202)**	**2.16**	**81.5**
$(\bar{2}\bar{3}0)$	2.21	2.3
$(\bar{2}30)$	2.21	2.4
$(2\bar{3}0)$	2.21	2.4
(230)	2.21	2.3
$(\bar{1}\bar{4}1)$	2.40	3.9
$(\bar{1}4\bar{1})$	2.40	4.1
$(1\bar{4}\bar{1})$	2.40	3.9
(141)	2.40	4.1

Table 3: Miller indices corresponding to peak 3 ($2.5 < Q < 2.8$ [1/Å]), as labeled in Figure 2(a) of the main text. Peaks are shown with increasing value of Q. Reflections below 2 % of I_{max} are not reported.

Indices	Q [1/Å]	I/I_{max} [%]
$(\bar{2}40)$	2.63	16.0
(240)	2.63	16.6
(042)	2.63	15.8
$(04\bar{2})$	2.63	16.3
(321)	2.64	12.4
$(32\bar{1})$	2.64	12.9
$(3\bar{2}1)$	2.64	12.6
$(\bar{3}21)$	2.64	13.1
$(\bar{1}\bar{2}3)$	2.65	18.3
$(1\bar{2}3)$	2.65	17.5
$(1\bar{2}\bar{3})$	2.65	18.4
(123)	2.65	17.4

Table 4: Miller indices corresponding to peak 4 ($2.85 < Q < 3.30$ [1/Å]), as labeled in Figure 2(a) of the main text. Peaks are shown with increasing value of Q. Reflections below 2 % of I_{max} are not reported. The reflections highlighted in bold constitute the vast majority of the signal measured in peak 4.

Indices	Q [1/Å]	I/I_{max} [%]
$\mathbf{(242)}$	**3.04**	**34.7**
$\mathbf{(2\bar{4}\bar{2})}$	**3.04**	**36.7**
$\mathbf{(\bar{2}4\bar{2})}$	**3.04**	**35.3**
$\mathbf{(\bar{2}\bar{4}2)}$	**3.04**	**35.6**
$\mathbf{(400)}$	**3.05**	**27.9**
$\mathbf{(004)}$	**3.07**	**27.9**
(401)	3.14	4.4
$(\bar{4}01)$	3.14	3.4
$(10\bar{4})$	3.16	3.4
(104)	3.16	4.4
(143)	3.23	3.3
$(1\bar{4}\bar{3})$	3.23	2.8
$(\bar{1}4\bar{3})$	3.23	3.4
$(\bar{1}\bar{4}3)$	3.23	2.8
(341)	3.23	5.1
$(3\bar{4}\bar{1})$	3.23	4.8
$(\bar{3}4\bar{1})$	3.23	5.3
$(\bar{3}\bar{4}1)$	3.23	4.6

Table 5: Miller indices and simulated intensities corresponding to peak 5 ($3.45 < Q < 3.85$ [1/Å]), as labeled in Figure 2(a) of the main text. Peaks are shown with increasing value of Q. Reflections below 2 % of I_{max} are not reported. The reflections highlighted in bold constitute the vast majority of the signal measured in peak 4.

Indices	Q [1/Å]	I/I_{max} [%]
(440)	3.73	14.5
($4\bar{4}0$)	3.73	15.1
($0\bar{4}4$)	3.74	14.5
(044)	3.74	14.7

References

(1) Stoumpos, C. C.; Malliakas, C. D.; Peters, J. A.; Liu, Z.; Sebastian, M.; Im, J.; Chasapis, T. C.; Wibowo, A. C.; Chung, D. Y.; Freeman, A. J.; Wessels, B. W.; Kanatzidis, M. G. Crystal Growth of the Perovskite Semiconductor CsPbBr3: A New Material for High-Energy Radiation Detection. *Crystal Growth & Design* **2013**, *13*, 2722–2727.

(2) Persson, K. Materials Data on CsPbBr3 (SG:221) by Materials Project. 2014; An optional note.

(3) Persson, K. Materials Data on PbBr2 (SG:62) by Materials Project. 2014; An optional note.

(4) Zhao, M.; Shi, Y.; Dai, J.; Lian, J. Ellipsometric study of the complex optical constants of a CsPbBr3 perovskite thin film. **2018**, *6*, 10450–10455.

(5) Yan, W.; Mao, L.; Zhao, P.; Mertens, A.; Dottermusch, S.; Hu, H.; Jin, Z.; Richards, B. S. Determination of complex optical constants and photovoltaic device design of all-inorganic CsPbBr3 perovskite thin films. **2020**, *28*, 15706.

(6) Evarestov, R. A.; Kotomin, E. A.; Senocrate, A.; Kremer, R. K.; Maier, J. First-principles comparative study of perfect and defective CsPbX3 (X = Br, I) crystals. **2020**, *22*, 3914–3920.

(7) Persson, K. Materials Data on CsPbBr3 (SG:62) by Materials Project. 2014; An optional note.

(8) Protesescu, L.; Yakunin, S.; Bodnarchuk, M. I.; Krieg, F.; Caputo, R.; Hendon, C. H.; Yang, R. X.; Walsh, A.; Kovalenko, M. V. Nanocrystals of Cesium Lead Halide Perovskites (CsPbX3, X = Cl, Br, and I): Novel Optoelectronic Materials Showing Bright Emission with Wide Color Gamut. *Nano Letters* **2015**, *15*, 3692–3696.

(9) Kittel, C. *Introduction to Solid State Physics*, 8th ed.; Wiley, 2004.

(10) Wu, X. et al. Light-induced picosecond rotational disordering of the inorganic sublattice in hybrid perovskites. *Science Advances* **2017**, *3*, e160238.

(11) López, C. A.; Abia, C.; Alvarez-Galván, M. C.; Hong, B.-K.; Martínez-Huerta, M. V.; Serrano-Sánchez, F.; Carrascoso, F.; Castellanos-Gómez, A.; Fernández-Dı
az, M. T.; Alonso, J. A. Crystal Structure Features of CsPbBr3 Perovskite Prepared by Mechanochemical Synthesis. *ACS Omega* **2020**, *5*, 5931–5938.

(12) Peng, L.; Dudarev, S.; Whelan, M. *High-Energy Electron Diffraction and Microscopy*; Oxford University Press, 2004.

(13) Elbaz, G. A.; Ong, W.-L.; Doud, E. A.; Kim, P.; Paley, D. W.; Roy, X.; Malen, J. A. Phonon Speed, Not Scattering, Differentiates Thermal Transport in Lead Halide Perovskites. *Nano Letters* **2017**, *17*, 5734–5739.
